# Most Random Gene Expression Signatures Are Significantly Associated with Breast Cancer Outcome

**DOI:** 10.1371/journal.pcbi.1002240

**Published:** 2011-10-20

**Authors:** David Venet, Jacques E. Dumont, Vincent Detours

**Affiliations:** 1 IRIDIA-CoDE, Université Libre de Bruxelles (U.L.B.), Brussels, Belgium; 2 IRIBHM, Université Libre de Bruxelles (U.L.B.), Campus Erasme, Brussels, Belgium; 3 WELBIO, Université Libre de Bruxelles (U.L.B.), Campus Erasme, Brussels, Belgium; Jefferson Medical College/Thomas Jefferson University, United States of America

## Abstract

Bridging the gap between animal or *in vitro* models and human disease is essential in medical research. Researchers often suggest that a biological mechanism is relevant to human cancer from the statistical association of a gene expression marker (a signature) of this mechanism, that was discovered in an experimental system, with disease outcome in humans. We examined this argument for breast cancer. Surprisingly, we found that gene expression signatures—unrelated to cancer—of the effect of postprandial laughter, of mice social defeat and of skin fibroblast localization were all significantly associated with breast cancer outcome. We next compared 47 published breast cancer outcome signatures to signatures made of random genes. Twenty-eight of them (60%) were not significantly better outcome predictors than random signatures of identical size and 11 (23%) were worst predictors than the median random signature. More than 90% of random signatures >100 genes were significant outcome predictors. We next derived a metagene, called meta-PCNA, by selecting the 1% genes most positively correlated with proliferation marker PCNA in a compendium of normal tissues expression. Adjusting breast cancer expression data for meta-PCNA abrogated almost entirely the outcome association of published and random signatures. We also found that, in the absence of adjustment, the hazard ratio of outcome association of a signature strongly correlated with meta-PCNA (R^2^ = 0.9). This relation also applied to single-gene expression markers. Moreover, >50% of the breast cancer transcriptome was correlated with meta-PCNA. A corollary was that purging cell cycle genes out of a signature failed to rule out the confounding effect of proliferation. Hence, it is questionable to suggest that a mechanism is relevant to human breast cancer from the finding that a gene expression marker for this mechanism predicts human breast cancer outcome, because most markers do. The methods we present help to overcome this problem.

## Author Summary

Proving that research findings from *in vitro* or animal models are relevant to human diseases is a major bottleneck in medical science. Hundreds of researchers have suggested the human relevance of oncogenic mechanisms from the statistical association between gene expression markers of these mechanisms and disease outcome. Such evidence has become easier to obtain recently with the advent of microarray screens and of large public-domain genome-wide expression datasets with patient follow-up. We demonstrated that in breast cancer any set of 100 genes or more selected at random has a 90% chance to be significantly associated with outcome. Thus, investigators are bound to find an association however whimsical their marker is. For example, we could establish outcome associations for a signature of postprandial laughter and a signature of social defeat in mice. Association was not stronger than expected at random for 28 (60%) of 47 published breast cancer signatures. The odds of association are 5–17% with random single gene markers—a finding relevant to older breast cancer studies. We explained these results by showing that much of the breast cancer transcriptome is correlated with proliferation, which integrates most prognostic information in this disease.

## Introduction

Ethics limits experimental investigation on human subjects. Hence, most experimental biomedical research is performed on animal and/or *in vitro* models. Proving that findings from model systems are relevant to human health is a major bottleneck.

Hundreds of studies in oncology have suggested the biological relevance to human of putative cancer-driving mechanisms with the following three steps: 1) characterize the mechanism in a model system, 2) derive from the model system a marker whose expression changes when the mechanism is altered, and 3) show that marker expression correlates with disease outcome in patients—the last figure of such paper is typically a Kaplan-Meier plot illustrating this correlation.

Breast cancer has been a test bed in oncogenomics. Several landmark studies (reviewed in ref. [1]) uncovered multi-gene mRNA markers of disease recurrence, which are independent of classical clinical markers and may provide useful information to guide treatment. These clinically motivated multi-genes markers, also called signatures, were derived from compendia of genome-wide mRNA tumoral profiles by selecting genes whose expression correlated with outcome [2]–[5], or with known aggressiveness markers such as proliferation [6]–[9] or grade [10]–[12].

Beyond clinical utility, many signatures were derived as markers of specific mechanisms and/or biological states and their association with outcome was evaluated in the context of studies structured along the 3-steps outlined above. These include signatures of stem cells [13]–[15], aneuploidy [16], wound healing [17], [18], hypoxia [19], [20], stromal component [21], epithelial-mesenchymal transition [22]–[24]; of mutations in TP53 [25], ALK5 [26]; of loss of PTEN [27]; of perturbations of E2F1 [28], bromodomain 4 [29], mir31 targets [30], p18^ink4c^ [31], retinoic acid receptor [32]; of anchorage-independent growth [33], activation of modules related to the proteasome and mitochondrions [34], etc. Contrasting with this diversity, meta-analyses of several outcome signatures have shown that they have essentially equivalent prognostic performances [35], [36], and are highly correlated with proliferation [7]–[8], [37], a predictor of breast cancer outcome that has been used for decades [38]–[40].

This raises a question: are all these mechanisms major independent drivers of breast cancer progression, or is step #3 inconclusive because of a basic confounding variable problem? To take an example of complex system outside oncology, let us suppose we are trying to discover which socio-economical variables drive people's health. We may find that the number of TV sets per household is positively correlated with longer life expectancy. This, of course, does not imply that TV sets improve health. Life expectancy and TV sets per household are both correlated with the gross national product per capita of nations, as are many other causes or byproducts of wealth such as energy consumption or education. So, is the significant association of say, a stem cell signature, with human breast cancer outcome informative about the relevance of stem cells to human breast cancer?

Resolving this issue has become more pressing recently. Several large cohorts with genome-wide tumoral expression profiles and patient follow-ups are available in the public domain. Servers resting on these data [41], [42] make step #3 accessible to anyone with an Internet connection. Genome-wide expression profiling has also considerably lowered the barrier to step #2. The search for markers is reduced to a nearly automated screen by comparing microarray profiles in situations where the putative cancer-driving mechanism is active or inactive. The end result is an increasing number of signatures.

Few studies using the outcome-association argument present negative controls to check whether their signature of interest is indeed more strongly related to outcome than signatures with no underlying oncological rationale. In statistical terms, these studies typically rest on H_0_ assuming a background of no association with outcome. The negative controls we present here prove this assumption wrong: a random signature is more likely to be correlated with breast cancer outcome than not. The statistical explanation for this phenomenon lies in the correlation of a large fraction of the breast transcriptome with one variable, we call it meta-PCNA, which integrates most of the prognostic information available in current breast cancer gene expression data.

## Results

### Most signatures not biologically related to cancer are statistically associated with breast cancer outcome

**Figure 1:**
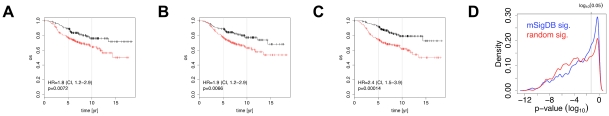
Association of negative control signatures with overall survival. In plots A–C the NKI cohort was split into two groups using a signature of post-prandial laughter (panel A), localization of skin fibroblasts (panel B), social defeat in mice (panel C). In panels A–C, the fraction of patients alive (overall survival, OS) is shown as a function of time for both groups. Hazard ratios (HR) between groups and their associated p-values are given in bottom-left corners. Panel D depicts p-values for association with outcome for all MSigDB c2 signatures and random signatures of identical size as MSigDB c2 signatures.

In order to assess whether association with outcome was specific, we tested the association with breast cancer outcome of three signatures whose rationale does not suggest any connection with cancer: a signature of the effect of postprandial laughter on peripheral blood mononuclear cells [43], a signature of skin fibroblast localization [44] and a signature of social defeat obtained from mice brains [45]. For the sake of simplicity, and because this is the most commonly used setup in the field, we focused on the 295 patients of the Netherlands Cancer Institute (a.k.a. NKI) cohort [2] and the overall survival end-point. Details on the procedure used to estimate association with outcome are provided in Supporting Information (Text S1). Surprisingly, the three control signatures were significantly associated with outcome (Figure 1, panels A–C).

To check that these were not anecdotal observations, we downloaded all signatures from MSigDB database [46] belonging to the c2 category and assessed their association with outcome. MSigDB c2 signatures are manually curated from the literature on gene expression and also include gene sets from curated pathways databases such as KEGG. Trivial single-gene signatures were removed. The 1890 signatures examined in MSigDB c2 encompass all the fields of biomedical sciences, nevertheless we discovered that 67% of them were associated with breast cancer outcome at p<0.05, 23% at p<10^−5^ (Figure 1D).

Cancer is a major subject matter of biomedical research, thus MSigDB c2 may be enriched for cancer-related signatures. To rule out the potential effect of a cancer bias, we generated for each signature in MSigDB c2 a signature of identical size but selected its genes randomly in the human genome. Although they are completely devoid of any biological rationale, 77% of these signatures were associated with outcome at p<0.05, and 30% at p<10^−5^ (Figure 1D).

Thus, nominal p-values should not be used directly because a signature associated with outcome with a significance of 10^−5^ and even more so, 0.05, is not more related to outcome than a random set of genes.

### Most published breast cancer signatures are not more strongly associated with breast cancer outcome than sets of random genes

Although most random signatures are significantly associated with breast cancer outcome, the association could be much stronger for published breast cancer signatures and provide valid statistical support for their relevance.

**Figure 2:**
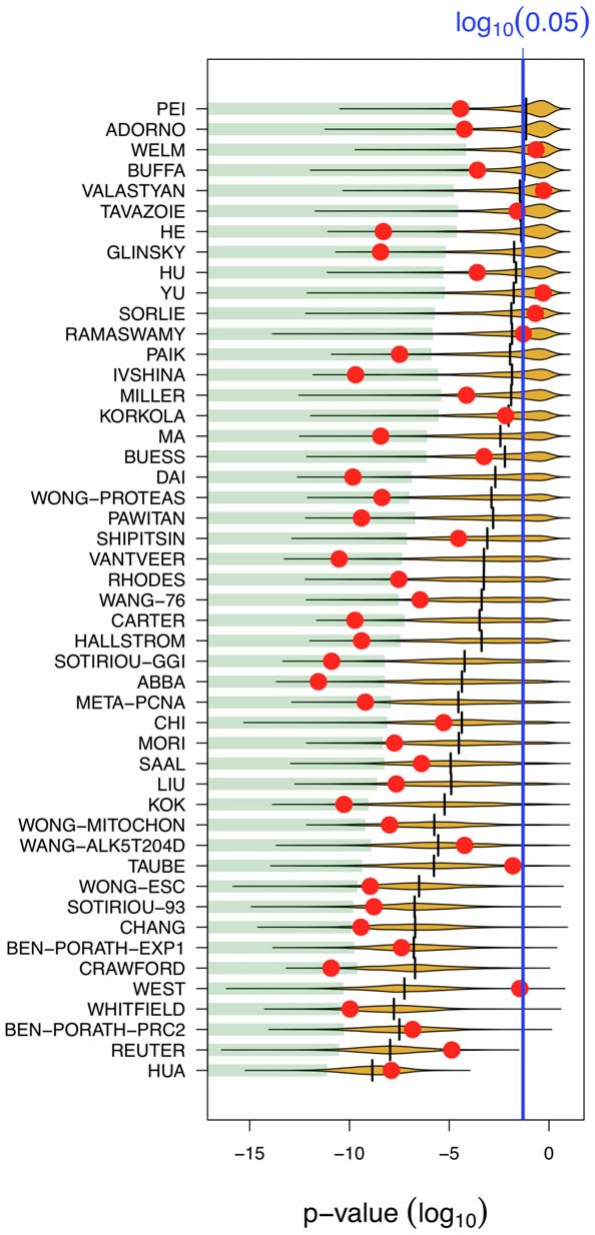
Most published signatures are not significantly better outcome predictors than random signatures of identical size. The x-axis denotes the p-value of association with overall survival. Red dots stand for published signatures, yellow shapes depict the distribution of p-values for 1000 random signatures of identical size, with the lower 5% quantiles shaded in green and the median shown as black line. Signatures are ordered by increasing sizes.

We compiled 47 signatures from the literature. Association with outcome has been reported for most of them (Supporting Information, Text S1), either for the purpose of finding better prognostic tools, or, in most cases, to suggest biological relevance. We compared the outcome association of each signature to that of 1000 random signatures of identical size (Figure 2). We confirmed the outcome association of 42 in these 47 signatures. Yet, 11 of them (23%) showed a weaker association than the median of random signatures. Abiding to statistical standard, one may consider a signature biologically relevant if its association with outcome is stronger than the association of the best 5% random signatures. Only 18 signatures in 47 (40%) met this criterion.

Figure 2 reveals that larger signatures are more significant. More than 90% of the signatures >100 genes we generated were significant at p<0.05. For the two largest ones, 714 and 1345 genes respectively, all 1000 random signatures tested were significant.

At the other end of the size spectrum, we found that 26% of individual genes printed on the NKI arrays were associated with outcome at p<0.05. Thus, a single gene study has 26 chances in 100 to yield a significant association. When we applied a q-value correction [47]—relevant to genome-wide studies—17% of all genes were associated with outcome at q<0.05. A comparable calculation was presented by Ein-Dor et al. [48]: 1234 genes among 5852 that passed their initial filter were associated with outcome with a false discovery rate <10%.

### Meta-PCNA integrates most of the outcome-related signal contained in the breast cancer transcriptome

Proliferation is a well-known breast cancer prognostic marker [38]–[40]. Cycling cells express thousands of specific genes [49], thus genome-wide expression profiles are likely to capture the fraction of cycling cells within a tissue. A proliferation cluster was noticed in early breast cancer microarray studies [50]–[52], and proliferation is the major variable behind gene expression-based breast cancer prognosis [7]–[9]. We devised a new metagene, meta-PCNA, in order to investigate further the role of proliferation.

The proliferating cell nuclear antigen, PCNA, is a ring-shaped protein that encircles DNA and regulates several processes leading to DNA replication [53]. As suggested by its name, this is one of the most widely used antigen target for immunohistochemical measures of the fraction of proliferating cells in tissues. Ge *et al.* [54] profiled with microarrays 36 tissues from normal, healthy, individuals encompassing 27 organs. We call ‘meta-PCNA’ the signature composed of the 1% genes the most positively correlated with PCNA expression across these 36 tissues (Table S1). In plain language, meta-PCNA genes are consistently expressed when PCNA is expressed in normal tissues and consistently repressed when PCNA is repressed. We define the meta-PCNA index as the median expression of meta-PCNA genes. Beside PCNA itself, meta-PCNA includes other canonical proliferation markers such as MKI67, TOP2A, MCM2, etc.

**Figure 3:**
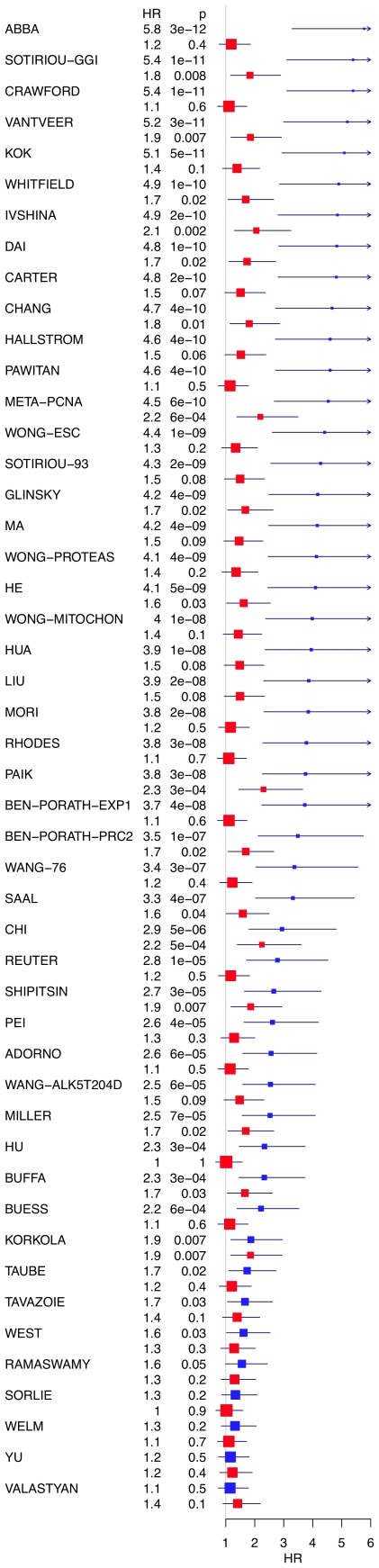
Meta-PCNA adjustment decreases the prognostic abilities of published signatures. Hazard ratios for overall survival association of 48 signatures in the original dataset (blue) and the meta-PCNA-adjusted dataset (red). Box sizes are inversely related to the size of the confidence intervals. Related Kaplan-Meier plots are available in the Supporting Information (Text S1).

We next compared for each one of the 47 published signatures its association with outcome in the original NKI data set and after adjustment of expression levels for the meta-PCNA index (Figure 3, Kaplan-Meier plots in Supporting Information, Text S1). Their association with outcome dropped dramatically after adjustment, although a few signatures remained strongly outcome associated. Any transformation damaging expression data will trivially decrease the association between outcome and expression. To control that was not the case with our adjustment procedure we reran the same analysis, except that meta-PCNA values were permuted randomly among patients prior to adjustment. In contrast with the adjustment of the actual non-permuted index, outcome association was not affected (Supporting Information, Text S1).

**Figure 4:**
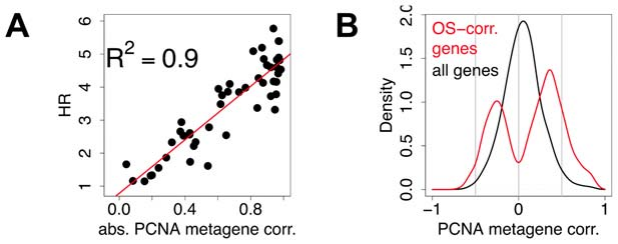
Most prognostic transcriptional signals are correlated with meta-PCNA. A) Each point denotes a signature. The x-axis depicts the absolute value of the correlation of the first principal component of the signatures with meta-PCNA, the y-axis depicts the hazard ratio for outcome association. Details of the analysis for each data point are available in the Supporting Information (Text S1). B) Distribution of the correlations of individual genes with meta-PCNA, for genes significantly associated with overall survival (red) and for all the genes spotted on the microarrays (black).

We plotted the hazard ratios of the 47 signatures against the absolute correlation of their first principal component with the meta-PCNA index. The more a signature was correlated with meta-PCNA, the higher its hazard ratio (R^2^ = 0.9, Figure 4A, details for each data point in Supporting Information, Text S1).

Since only a limited set of genes is included in the 47 signatures, we plotted the distribution of correlations with the meta-PCNA index of all genes significantly associated with outcome and, as a negative control, of all genes printed on the microarrays (Figure 4B). Among the 17% of genes associated with outcome at *q*<0.05, 91% were significantly correlated with meta-PCNA. Thus, any predictor resting on a linear combination of genes associated with outcome has a high probability to be confounded by proliferation.

### More than 50% of the breast cancer transcriptome is correlated with meta-PCNA, hence removing cell cycle genes from a signature cannot rule out proliferation as a confounder

The potential confounding effect of proliferation has been recognized by a number of authors who attempted to rule it out by removing known proliferation genes from expression data [17], [14], [15]. These genes have been defined in various ways, including the Gene Ontology ‘cell cycle’ category, the genes periodically regulated in a cell-cycle time course [49], or genes of the breast cancer ‘proliferation cluster’ [55].

**Figure 5:**
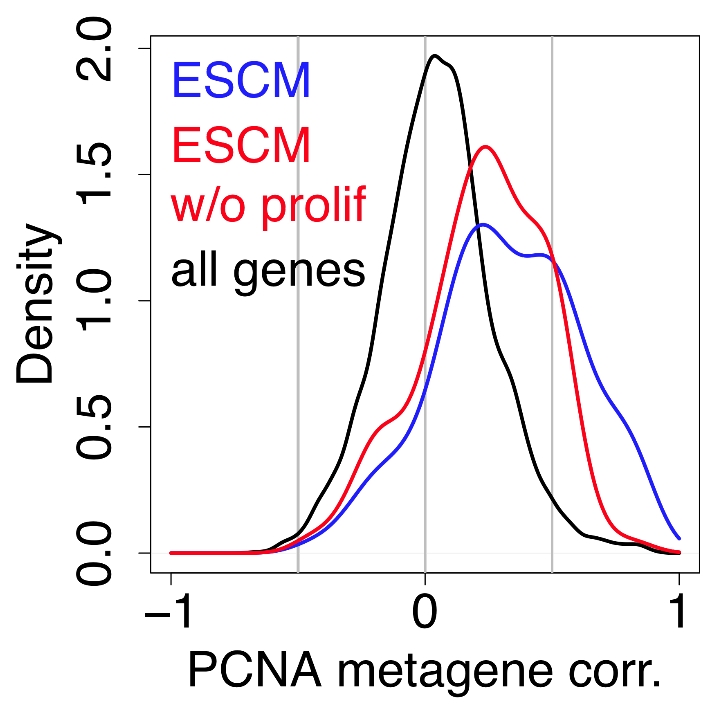
Purging cell cycle genes from a signature does not rule out proliferation signals. Distribution of the correlations with meta-PCNA of genes in the Embryonic Stem Cell Module (blue, ref. [15]), of the correlations of the same module with its cell cycle genes removed (red) and of all of the genes spotted on the microarray (black).

Following Ben Porath et al. [14], we defined as cell-cycle genes any gene present in at least one of these three categories. We calculated the distributions of correlations between the meta-PCNA index and genes of the Embryonic Stem Cell Module (ESCM) of Wong et al. [15], with and without the cell cycle genes (Figure 5). Purging these genes out of the ESCM did eliminate signals in the highest correlation range, but the ESCM remained vastly more correlated with meta-PCNA than the bulk of genes printed on the arrays (p = 10^−25^).

Moreover, 58% of the genes printed on the array were significantly correlated with the meta-PCNA index in the NKI cohort. Thus, the correlations with meta-PCNA extend far beyond cell cycle genes. Removing these genes fails to rule out the confounding effect of proliferation. Similarly, a signature does not have to be enriched with known cell cycle genes to convey a strong cell proliferation signal.

### Results are reproducible across cohorts and end-points

Previous sections rested on the NKI data set and the overall survival end-point. Are our observations specific of this popular, but not universal, setup? We reran the analyses using recurrence-free survival, and on another cohort [56] using both overall survival and relapse-free survival.

**Figure 6:**
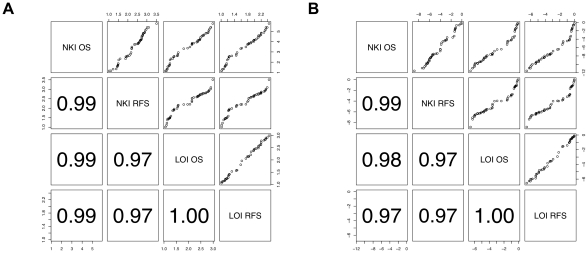
Reproducible outcome predictions across end-points and cohorts. Each dot represents a published signature. A) Hazard ratios. B) Log rank p-values. Lower panels give correlation coefficients for corresponding scatter plots in the symmetric upper panels. OS, overall survival; RFS, recurrence-free survival. NKI, data from ref. [2]; LOI, data from ref. [56].

We calculated hazard ratios for the 47 published signatures using all combinations of end-points and cohorts. Correlation between hazard ratios among the different cohorts/end-points was ≥0.97 (Figure 6). Thus, the ranking of the signatures with respect to association with outcome was highly reproducible. However, the combination of NKI data and overall survival gave hazard ratios ∼1.3 units higher (median HR = 3.8 in NKI and OS vs. <2.5 in other setups). Accordingly, p-values were ∼4 orders of magnitude smaller than when association with outcome was estimated from the overall survival in the cohort of Loi et al. [56], although it included ∼30% more patients. This difference between the 2 cohorts is less marked with relapse-free survival. Nevertheless, our analysis (summarized Table 1) reveals that, irrespective of the specific setup, at least 40% of MSigDB c2 signatures and 5% of all genes are associated with outcome, and at most 40% of the 47 published signatures are better than the 5% best same-size random signatures.

**Table 1:** Summary of analysis with different cohorts and end points.

	NKI OS	NKI RFS	LOI OS	LOI RFS
Fraction of patient experiencing an event	79/295	101/295	96/380	139/393
% MSigDB c2 with p<0.05	67%	56%	52%	45%
% of all genes with p<0.05	17%	9%	8%	5%
% BC signatures better than 5% best random signatures of same size	40%	35%	29%	31%
Correlation of BC signatures HR with their association with meta-PCNA	0.9	0.9	0.9	0.9

## Discussion

There are many ways to estimate association between the expression of a multi-gene marker and disease outcome, and different studies have taken different routes. Our goal to compare signatures and assess them against negative controls, however, implied a uniform statistical framework. We present a comparison of a number of such methods in the Supporting Information (Text S1). A popular approach used in the studies we reviewed consists in stratifying the patients by hierarchical clustering in the signature subspace [57], [21], [29], [24], [28], [15], [58]. In most cases, our method of choice (using the first component of a Principal Components Analysis of a signature as a prognostic score) reveals stronger outcome associations than this approach. Our method is validated by the fact that we could reproduce the outcome association of most published signatures, which, conversely, validates the prognostic value of those signatures. The choice of association method is of course important, as there is a possibility that it misses some signals captured by specific combinations of signatures and models. However, most papers use similarly simple methods as ours. Furthermore, the strength of such association might be doubted if it depended on an elaborate algorithm, as it is likely to be caused by spurious signals arising from model selection biases.

The main message of this paper is that, if the purpose of a study is to assert the biological relevance to human cancer of a signature, the association between this signature and outcome cannot rest on the nominal p-values, as obtained on breast cancer by the Cox analysis. This follows from elevated likelihood that random sets of genes are related to the outcome. Thus, an investigator finding that her/his signature is associated with outcome with a significance of 10^−5^, and even more so, 0.05, gains no specific information because sets of random genes would likely yield similar, or better, results. Nominal p-values do not answer the appropriate statistical question: the question is not whether a given set of genes is related to survival, but whether it is more related to survival than random sets of genes.

This problem extends to single-gene markers and therefore to many studies published in the pre-genomic era. Claims similar to those concerning signatures have been made, that single genes, important in a model system, are relevant for human cancer progression based on differential expression between short- and long-survival groups. As 26% of the genes are related to survival at p<0.05 (17% at q<0.05), much tighter p-values than commonly used should be imposed to demonstrate such a relation.

Several studies in the panel of 47 we investigated developed arguments independent of outcome association. For example, Hu et al. [59] used outcome association not as a validation argument, but as an exploratory tool to discover driver DNA copy number aberrations, which were then directly investigated. However, most of these studies, and many more not reviewed here, extrapolated the results from animal or highly artificial *in vitro* models to human *in vivo* cancer on the basis of questionable association statistics alone.

The present study addresses purely correlative association between gene-expression and disease outcome. We have shown that proliferation integrates most of the prognostic information contained in the breast cancer transcriptome. Yet—we cannot stress this enough—we have *not* shown that proliferation is a core driving force behind breast cancer progression. Disentangling the role of a biological process in cancer progression *in vivo* from the role of proliferation and from the role of the other processes associated with it is a crucial issue. The adjustment methodology we propose may be useful in assessing whether markers of biological processes do or do not rest on association with proliferation. Our results also imply that such markers should be evaluated against the outcome association of comparable negative control markers.

Our study questions the biological interpretation of the prognostic value of published breast cancer signatures, but has no bearing on their usefulness in the clinic: a marker may be accurate without yielding interesting biological insight regarding the mechanism of disease progression. Nevertheless, the prominence of proliferation should be taken into account in future clinical research. Are there transcriptional signals in breast cancer that are prognostic, but independent of proliferation? Is there any hope to perform better than the 70 genes NKI signature [2]? The studies we reviewed assessed outcome prediction from gene expression measured in bulk tumors sampled from a relatively wide spectrum of patients, thus prognostic transcriptional signals detectable in specific tumor cells and/or specific patient groups were out of scope. Yet, proliferation-related signals are prognostic mostly in ER+ tumors [1]. Immunological genes convey prognostic information in ER- tumors and in tumors with HER2 amplification [8], [60]–[64]. This information is unquestionably independent of proliferation since it improves prognostic accuracy beyond the abilities of proliferation-driven signatures and classical clinical markers [65]. Larger cohorts allowing the analysis of patients sub-groups and expression profiling of specific tumor cells/tumor areas may lead to better prognostic tools in the future.

In conclusion, we have shown that 1) random single- and multiple-genes expression markers have a high probability to be associated with breast cancer outcome; 2) most published signatures are not significantly more associated with outcome than random predictors; 3) the meta-PCNA metagene integrates most of the outcome-related information contained in the breast cancer transcriptome; 4) this information is present in over 50% of the transcriptome and cannot be removed by purging known cell-cycle genes from a signature.

## Methods

### Software setup

All analyses were run with R 2.9.0 [66] with packages specified in the following sections. Functions were run with default parameters unless specified otherwise.

### Code and data availability

The code and data underlying the results and figures of this study are available as a Bzip2-compressed tar bundle from the *PLoS Computational Biology* web site (Dataset S1, size is 87 MB). The scripts assume a UNIX/LINUX environment.

### Expression data

All the data were available from public sources:

- Ge *et al.* [54] data were downloaded from NCBI's Gene expression Omnibus (www.ncbi.nlm.nih.gov/geo; accession, GDS1096). We renormalized the raw data (CEL files) using Bioconductor [67] package gcrma [68].
- Loi *et al.* [56] data were downloaded from NCBI's Gene expression Omnibus (accession GSE6532). We used the Rdata file.
- The NKI, a.k.a. van de Vijver *et al.* [2], data set was downloaded from the Rosetta Inpharmatics web site on April 26^th^ 2007 (www.rii.com, this site is now defunct, the dataset is available in the supplementary code and data tar bundle). Probe annotations were reconstructed using Bioconductor [67] package annotate. Contigs not mapped to genes in the original data set were recovered as much as possible using the table ArrayNomenclature_contig_accession.xls, also on Rosetta web site. We used the original authors normalization, but ignored the flags.

Probes mapping to the same genes were averaged in each one of the three datasets.

### Literature signatures

Whenever possible, the signatures were compiled from the publications online supplementary tables. When not available, the gene symbols were automatically read with an optical character recognition system from the papers tables and figures. In rare instances, signatures were encoded manually and double-checked. Because gene names and symbols are changing over time, the gene symbols of all signature genes were updated to match the HUGO nomenclature and therefore maximize the match with microarray gene annotations. HUGO gene symbols and their older aliases were obtained from the file gene_info as available on May 9^th^ 2007 from the NCBI ftp server.

MSigBD 2.0 c2 signatures were downloaded as a *.gmt file from the Broad Institute page www.broadinstitute.org/gsea/msigdb/index.jsp.

### Meta-PCNA index

We computed the Pearson correlation between PCNA and all the genes in the Ge et al. [54] dataset and selected the 1% most positively correlated, i.e., 131 genes out of 13,077, to form the meta-PCNA signature (Table S1). The meta-PCNA index of a tissue was computed from its expression profile by taking the median expression of these genes.

### Adjusting data for the meta-PCNA index

The expression of each gene was fitted with R's ‘lm’ function and each expression measurement was substituted by the sum of its residual and its mean expression across the cohort.

### Association of signatures with outcome

In order to systematically compare the published signatures to random signatures and evaluate the relation between outcome association and meta-PCNA, we needed an outcome association estimation procedure that is robust and fully automated. We systematically compared three procedures and selected among them the most sensitive and stable one. This is described in Supporting Information (Text S3.), only the selected method is described here. It consists in computing the first principal component (PC1) of the signature (with R's prcomp) and then split the cohort according to the median of PC1. Probes mapping to the same gene were averaged and, following Ramaswamy et al. [57], data were median polished (R's medpolish) before the dimension reduction step.

## Supporting Information

Dataset S1 **Script and data underlying this paper (size is 87 MB, unpack with UNIX bunzip2 then ‘tar xvf’).**
(BZIP2)

Table S1 **The meta-PCNA signature.**
(PDF)

Text S1 **Supplementary Information.**
(PDF)
