# Supplementary material for: Most Random Gene Expression Signatures Are Significantly Associated with Breast Cancer Outcome
Source: PLoS Comput Biol. 2011 Oct 20;7(10):e1002240. doi: 10.1371/journal.pcbi.1002240 (PMC3197658; doi:10.1371/journal.pcbi.1002240)
Supplement: Table S1 — The meta-PCNA signature. (PDF) [file pcbi.1002240.s002.pdf]

The meta-PCNA signature

SYMBOL	GENENAME	Entrez ID	PCNA correlation
PCNA	proliferating cell nuclear antigen	5111	1
CKS2	CDC28 protein kinase regulatory subunit 2	1164	0.85
NUSAP1	nucleolar and spindle associated protein 1	51203	0.83
RRM2	ribonucleotide reductase M2 polypeptide	6241	0.83
ZWINT	ZW10 interactor	11130	0.83
PRC1	protein regulator of cytokinesis 1	9055	0.82
TFDP1	transcription factor Dp-1	7027	0.82
CCNA2	cyclin A2	890	0.81
CCNB1	cyclin B1	891	0.81
MELK	maternal embryonic leucine zipper kinase	9833	0.81
TPX2	TPX2, microtubule-associated, homolog (Xenopus laevis)	22974	0.81
BIRC5	baculoviral IAP repeat-containing 5 (survivin)	332	0.8
NCAPG2	non-SMC condensin II complex, subunit G2	54892	0.8
RFWD3	ring finger and WD repeat domain 3	55159	0.8
TACC3	transforming, acidic coiled-coil containing protein 3	10460	0.8
CDC2	cell division cycle 2, G1 to S and G2 to M	983	0.79
KIAA0101	KIAA0101	9768	0.79
MCM2	MCM2 minichromosome maintenance deficient 2, mitotin (S. cerevisiae)	4171	0.79
MCM5	MCM5 minichromosome maintenance deficient 5, cell division cycle 46 (S. cerevisiae)	4174	0.79
ASF1B	ASF1 anti-silencing function 1 homolog B (S. cerevisiae)	55723	0.78
CCNB2	cyclin B2	9133	0.78
GTPBP2	GTP binding protein 2	54676	0.78
KIF20A	kinesin family member 20A	10112	0.78
PTTG1	pituitary tumor-transforming 1	9232	0.78
AURKA	aurora kinase A	6790	0.77
CDC20	cell division cycle 20 homolog (S. cerevisiae)	991	0.77
DKFZp762E1312	hypothetical protein DKFZp762E1312	55355	0.77
FOXM1	forkhead box M1	2305	0.77
GINS2	GINS complex subunit 2 (Psf2 homolog)	51659	0.77
MAD2L1	MAD2 mitotic arrest deficient-like 1 (yeast)	4085	0.77
UBE2C	ubiquitin-conjugating enzyme E2C	11065	0.77
GINS1	GINS complex subunit 1 (Psf1 homolog)	9837	0.76
MCM6	minichromosome maintenance deficient 6 homolog (S. cerevisiae)	4175	0.76
NCAPD2	non-SMC condensin I complex, subunit D2	9918	0.76
NUP37	nucleoporin 37kDa	79023	0.76
CKS1B	CDC28 protein kinase regulatory subunit 1B	1163	0.75
LOC146909	hypothetical protein LOC146909	146909	0.75
MCM7	MCM7 minichromosome maintenance deficient 7 (S. cerevisiae)	4176	0.75
ARID3A	AT rich interactive domain 3A (BRIGHT-like)	1820	0.74
AURKB	aurora kinase B	9212	0.74
CDCA8	cell division cycle associated 8	55143	0.74
SNRPD1	small nuclear ribonucleoprotein D1 polypeptide 16kDa	6632	0.74
TROAP	trophinin associated protein (tastin)	10024	0.74
C21orf45	chromosome 21 open reading frame 45	54069	0.73
DDX39	DEAD (Asp-Glu-Ala-Asp) box polypeptide 39	10212	0.73
ERAF	erythroid associated factor	51327	0.73
ESPL1	extra spindle pole bodies homolog 1 (S. cerevisiae)	9700	0.73
HMBS	hydroxymethylbilane synthase	3145	0.73
LSM6	LSM6 homolog, U6 small nuclear RNA associated (S. cerevisiae)	11157	0.73
MCM4	MCM4 minichromosome maintenance deficient 4 (S. cerevisiae)	4173	0.73
NCAPD3	non-SMC condensin II complex, subunit D3	23310	0.73
PPIH	peptidylprolyl isomerase H (cyclophilin H)	10465	0.73

CDC45L	CDC45 cell division cycle 45-like (S. cerevisiae)	8318	0.72
DTL	denticleless homolog (Drosophila)	51514	0.72
EPB42	erythrocyte membrane protein band 4.2	2038	0.72
HMGN2	high-mobility group nucleosomal binding domain 2	3151	0.72
MCM3	MCM3 minichromosome maintenance deficient 3 (S. cerevisiae)	4172	0.72
PF4	platelet factor 4 (chemokine (C-X-C motif) ligand 4)	5196	0.72
PPBP	pro-platelet basic protein (chemokine (C-X-C motif) ligand 7)	5473	0.72
RFC4	replication factor C (activator 1) 4, 37kDa	5984	0.72
TOP2A	topoisomerase (DNA) II alpha 170kDa	7153	0.72
BUB1B	BUB1 budding uninhibited by benzimidazoles 1 homolog beta (yeast)	701	0.71
CDT1	chromatin licensing and DNA replication factor 1	81620	0.71
FEN1	flap structure-specific endonuclease 1	2237	0.71
GATA1	GATA binding protein 1 (globin transcription factor 1)	2623	0.71
GYPB	glycophorin B (MNS blood group)	2994	0.71
HMGB2	high-mobility group box 2	3148	0.71
KIF22	kinesin family member 22	3835	0.71
KLF1	Kruppel-like factor 1 (erythroid)	10661	0.71
MLF1IP	MLF1 interacting protein	79682	0.71
RFC3	replication factor C (activator 1) 3, 38kDa	5983	0.71
RHCE	Rh blood group, CcEe antigens	6006	0.71
TAL1	T-cell acute lymphocytic leukemia 1	6886	0.71
TCF3	transcription factor 3 (E2A immunoglobulin enhancer binding factors E12/E47)	6929	0.71
ALAS2	aminolevulinate, delta-, synthase 2 (sideroblastic/hypochromic anemia)	212	0.7
BZRPL1	benzodiazapine receptor (peripheral)-like 1	222642	0.7
CDCA3	cell division cycle associated 3	83461	0.7
CENPA	centromere protein A	1058	0.7
CKLF	chemokine-like factor	51192	0.7
GTSE1	G-2 and S-phase expressed 1	51512	0.7
NFE2	nuclear factor (erythroid-derived 2), 45kDa	4778	0.7
OIP5	Opa interacting protein 5	11339	0.7
SHCBP1	SHC SH2-domain binding protein 1	79801	0.7
SNF8	SNF8, ESCRT-II complex subunit, homolog (S. cerevisiae)	11267	0.7
SNRPB	small nuclear ribonucleoprotein polypeptides B and B1	6628	0.7
SPTA1	spectrin, alpha, erythrocytic 1 (elliptocytosis 2)	6708	0.7
KEL	Kell blood group, metallo-endopeptidase	3792	0.69
KIF2C	kinesin family member 2C	11004	0.69
LYL1	lymphoblastic leukemia derived sequence 1	4066	0.69
PSMD9	proteasome (prosome, macropain) 26S subunit, non-ATPase, 9	5715	0.69
RACGAP1	Rac GTPase activating protein 1	29127	0.69
RPIA	ribose 5-phosphate isomerase A (ribose 5-phosphate epimerase)	22934	0.69
TIMELESS	timeless homolog (Drosophila)	8914	0.69
TRMT5	TRM5 tRNA methyltransferase 5 homolog (S. cerevisiae)	57570	0.69
TYMS	thymidylate synthetase	7298	0.69
VRK1	vaccinia related kinase 1	7443	0.69
FBXO7	F-box protein 7	25793	0.68
H3F3A	H3 histone, family 3A	3020	0.68
NUDT1	nudix (nucleoside diphosphate linked moiety X)-type motif 1	4521	0.68
PLEK	pleckstrin	5341	0.68
POLE2	polymerase (DNA directed), epsilon 2 (p59 subunit)	5427	0.68
RHAG	Rh-associated glycoprotein	6005	0.68
WHSC1	Wolf-Hirschhorn syndrome candidate 1	7468	0.68
APOBEC3B	apolipoprotein B mRNA editing enzyme, catalytic polypeptide-like 3B	9582	0.67
BPGM	2,3-bisphosphoglycerate mutase	669	0.67
CHAF1A	chromatin assembly factor 1, subunit A (p150)	10036	0.67

DNAJC9	DnaJ (Hsp40) homolog, subfamily C, member 9	23234	0.67
FBXO5	F-box protein 5	26271	0.67
KIF4A	kinesin family member 4A	24137	0.67
KLF15	Kruppel-like factor 15	28999	0.67
MKI67	antigen identified by monoclonal antibody Ki-67	4288	0.67
PGD	phosphogluconate dehydrogenase	5226	0.67
RPA3	replication protein A3, 14kDa	6119	0.67
SFRS2	splicing factor, arginine/serine-rich 2	6427	0.67
TRIM58	tripartite motif-containing 58	25893	0.67
ADAMTS13	ADAM metallopeptidase with thrombospondin type 1 motif, 13	11093	0.66
CDCA4	cell division cycle associated 4	55038	0.66
CDKN3	cyclin-dependent kinase inhibitor 3 (CDK2-associated dual specificity phosphatase)	1033	0.66
FECH	ferrochelatase (protoporphyria)	2235	0.66
LBR	lamin B receptor	3930	0.66
LIG1	ligase I, DNA, ATP-dependent	3978	0.66
LMNB1	lamin B1	4001	0.66
MICB	MHC class I polypeptide-related sequence B	4277	0.66
NUP210	nucleoporin 210kDa	23225	0.66
ORC6L	origin recognition complex, subunit 6 like (yeast)	23594	0.66
RAD51AP1	RAD51 associated protein 1	10635	0.66
RHD	Rh blood group, D antigen	6007	0.66
SMC4	structural maintenance of chromosomes 4	10051	0.66
GYPA	glycophorin A (MNS blood group)	2993	0.65
RPP30	ribonuclease P/MRP 30kDa subunit	10556	0.65
TRIM10	tripartite motif-containing 10	10107	0.65
