# Supplementary material for: Most Random Gene Expression Signatures Are Significantly Associated with Breast Cancer Outcome
Source: PLoS Comput Biol. 2011 Oct 20;7(10):e1002240. doi: 10.1371/journal.pcbi.1002240 (PMC3197658; doi:10.1371/journal.pcbi.1002240)
Supplement: Text S1 — Supplementary Information. (PDF) [file pcbi.1002240.s003.pdf]

Supplementary analysis for: Most random gene expression signatures are significantly associated with breast cancer outcome

David Venet, Jacques E. Dumont and Vincent Detours*

April 26, 2011

Contents

1 Additional methods and controls **2**

1.1 Estimating outcome association of multi-genes signatures . . 2

1.2 Adjusting expression for a permuted, nonsense, index does not impact outcome association . . . 3

2 Results for individual signatures **6**

2.1 Preliminaries . . . 6

2.2 ABBA . . . 6

2.3 ADORNO . . . 7

2.4 BEN-PORATH-EXP1 . . . 7

2.5 BEN-PORATH-PRC2 . . . 8

2.6 BUESS . . . 9

2.7 BUFFA . . . 9

2.8 CARTER . . . 10

2.9 CHANG . . . 11

2.10 CHI . . . 12

2.11 CRAWFORD . . . 13

2.12 DAI . . . 13

2.13 GLINSKY . . . 14

2.14 HALLSTROM . . . 15

2.15 HE . . . 15

2.16 HU . . . 16

2.17 HUA . . . 17

2.18 IVSHINA . . . 18

2.19 KOK . . . 19

*Correspondence to vdetours@ulb.ac.be

2.20 KORKOLA . . . 19
2.21 LIU . . . 20
2.22 MA . . . 21
2.23 MILLER . . . 22
2.24 MORI . . . 22
2.25 PAIK . . . 23
2.26 PAWITAN . . . 24
2.27 PEI . . . 24
2.28 RAMASWAMY . . . 25
2.29 REUTER . . . 26
2.30 RHODES . . . 27
2.31 SAAL . . . 28
2.32 SHIPITSIN . . . 28
2.33 SORLIE . . . 29
2.34 SOTIRIOU-93 . . . 30
2.35 SOTIRIOU-GGI . . . 31
2.36 META-PCNA . . . 32
2.37 TAUBE . . . 32
2.38 TAVAZOIE . . . 33
2.39 VALASTYAN . . . 33
2.40 VANTVEER . . . 34
2.41 WANG-76 . . . 35
2.42 WANG-ALK5T204D . . . 36
2.43 WELM . . . 37
2.44 WEST . . . 37
2.45 WHITFIELD . . . 38
2.46 WONG-ESC . . . 39
2.47 WONG-MITOCHON . . . 39
2.48 WONG-PROTEAS . . . 40
2.49 YU . . . 41

1 Additional methods and controls

1.1 Estimating outcome association of multi-genes signatures

In order to systematically compare the published signatures to random signatures and evaluate the relation between outcome association and meta-PCNA, we needed an outcome association estimation procedure that is robust and fully automated.

A number of studies in the 47 reviewed stratified good and bad prognosis groups according to the two main clusters obtained from hierarchical clustering (HC) applied to the genes in their signatures. This method can be automated. HC, however, is notoriously unstable.

We investigated two variants of the idea of stratifying the cohort with an unsupervised classification procedure. The first one is kmeans (R's `kmeans`), the second consists in computing the first principal component (PC1) of the signature (with R's prcomp) and then split the cohort according to the median of PC1.

HC was run as implemented by `hcluster` (`amap` R's package) with average linkage and the $1 - \text{correlation}$ distance.

The following preprocessing was applied before any of the three stratification steps. Probes mapping to the same gene were averaged and, following Ramaswamy et al. (Nat. Genet., 2003), data were median polished (R's `medpolish`).

Given a binary stratification of the cohort, the hazard ratio (HR) and the related log-rank p-values were computed with the standard Cox procedure implemented in R's `coxph`.

Which of HC, kmeans or PC1 is best? The method should be sensitive enough to detect associations with outcome. In addition, the outcome association of a signature should not change substantially if 10% of patients among the 295 patients of the van de Vijver et al. (NEJM, 2002) cohort are removed at random. Therefore, we estimated HR and p-values for all 47 signatures and the three dimension reduction methods across 1,000 runs with 10% of the samples randomly held aside. Estimates of HR and p-values are shown Figure S1 below.

Visual inspection of the boxplots suggests that PC1 yields higher hazard ratios and smaller log rank p-values, overall, than HC and even more so, kmeans. This higher sensitivity is confirmed ($p < 0.01$) by a robust multivariate analysis (`lmRob` from R's `robust` package) on the factors 'dimension reduction method' and 'signature'. PC1 also yields more stable estimates than HC. Therefore, we based our analysis on PC1.

1.2 Adjusting expression for a permuted, nonsense, index does not impact outcome association

Any procedure that damage the data would degrade its association with outcome. For example, replacing expression measures by random noise, or a bug in the adjustment script would have the effect presented in the forrest plot Fig. 3 in the main text. To control this was not occuring with our adjustment procedure we permuted the meta-PCNA index randomly among samples, and recalculated the forrest plot. Outcome association is preserved with this permuted, nonsense, index.

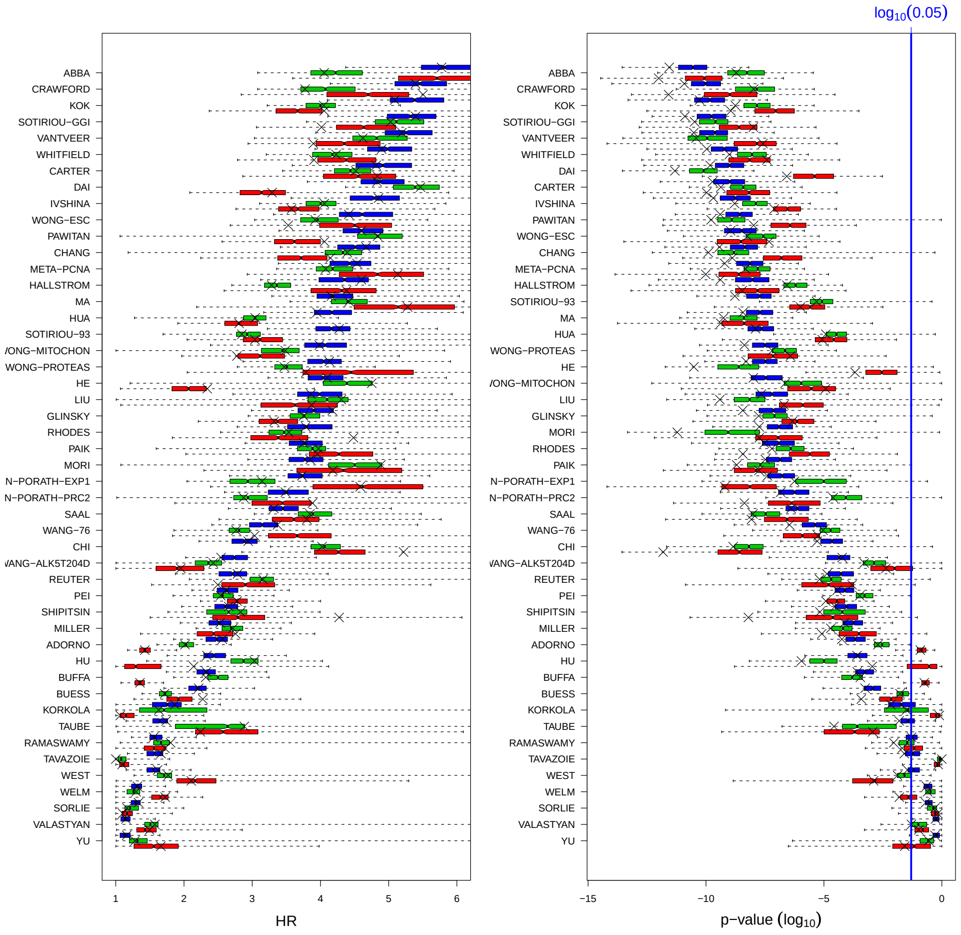

Figure 1: Hazard ratios (left) and log rank p-values (right) were computed for each signature and each dimension reduction methods over 1,000 versions of the cohort, each with 10% of the tumors removed at random. Red: HC; green: kmeans; blue: PC1; × symbols denote values for the full 295 tumors data set (p-values are in general slightly higher in the full data set due to increased statistical power). Boxes show the inter-quartile range and whiskers extreme values.

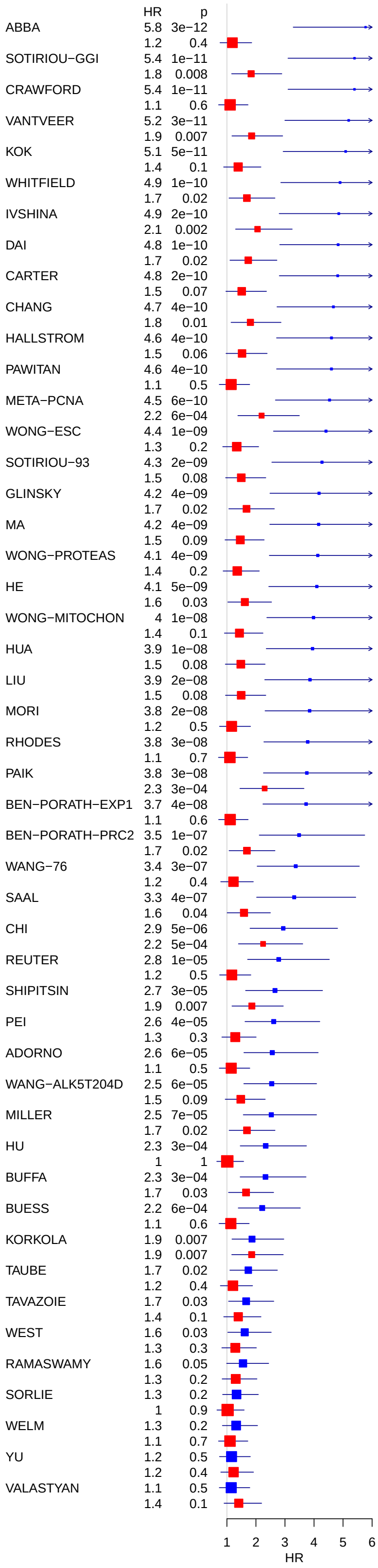

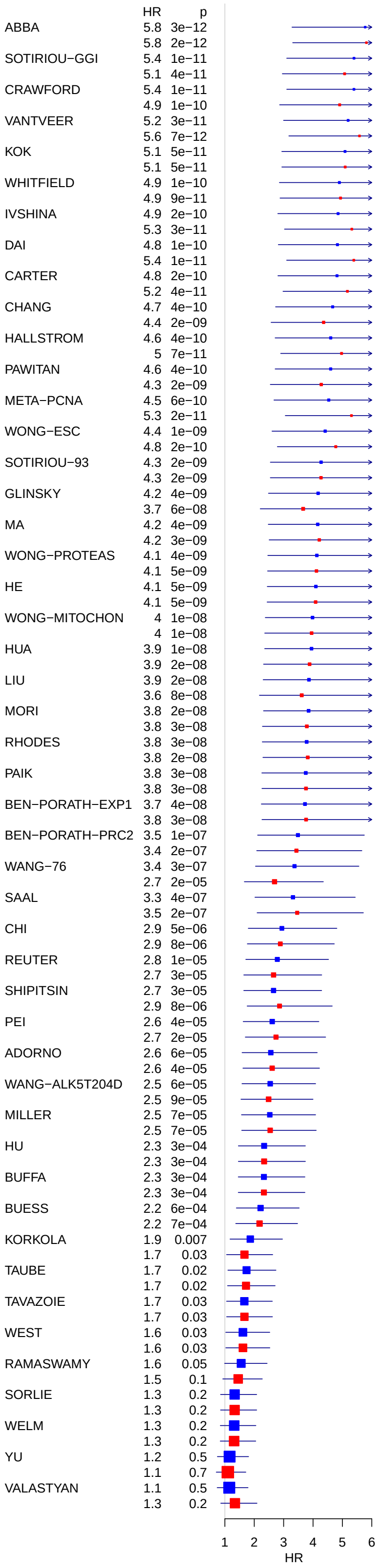

Figure 2: Left, forest plot for published signatures association with outcome in the original data (blue) and in data adjusted for the meta-PCNA index (red), it is Fig. 3 in the main text. Right, negative control: the data were adjusted with a permuted meta-PCNA index.

2 Results for individual signatures

2.1 Preliminaries

Graphics generated for each individual signatures are presented as follows. Left-hand side plots are Kaplan-Meier curves for outcome association estimated with the PC1 method described above. Center plots: same with expression data adjusted for the meta-PCNA index. Right-hand side plots, bottom panels: % of variance explained by the first 10 components of the signature (or $1-n$ if the signature includes $n < 10$ genes); top panels: correlation of individual components with meta-PCNA. Graphics in the top rows are for the NKI cohort, and Loi et al. cohort in the bottom row, overall survival is the end-point in both cases.

2.2 ABBA

- Title of publication: Breast cancer biomarker discovery in the functional genomic age: a systematic review of 42 gene expression signatures.
- Authors: MC Abba E Lacunza M Butti CM Aldaz
- Journal: Biomark Insights
- Date of publication: Month 2010
- PubMed ID: 21082037

111 genes symbols from this signature had aliases in Unigene. Among them, 106 are printed on the microarrays used here-in.

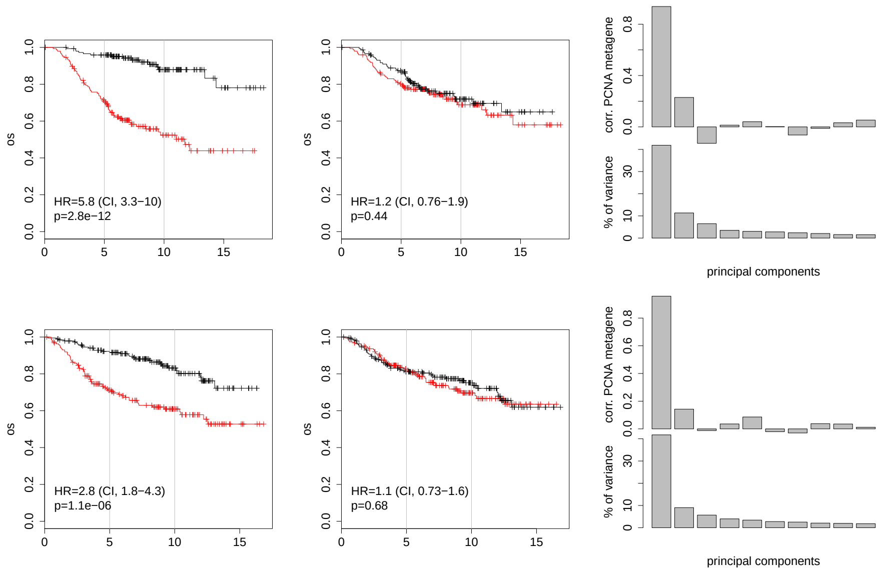

2.3 ADORNO

- Title of publication: A Mutant-p53/Smad complex opposes p63 to empower TGFbeta-induced metastasis.
- Authors: M Adorno M Cordenonsi M Montagner S Dupont C Wong B Hann A Solari S Bobisse MB Rondina V Guzzardo AR Parenti A Rosato S Bicciato A Balmain S Piccolo
- Journal: Cell
- Date of publication: Apr 2009
- PubMed ID: 19345189

2 genes symbols from this signature had aliases in Unigene. Among them, 2 are printed on the microarrays used here-in.

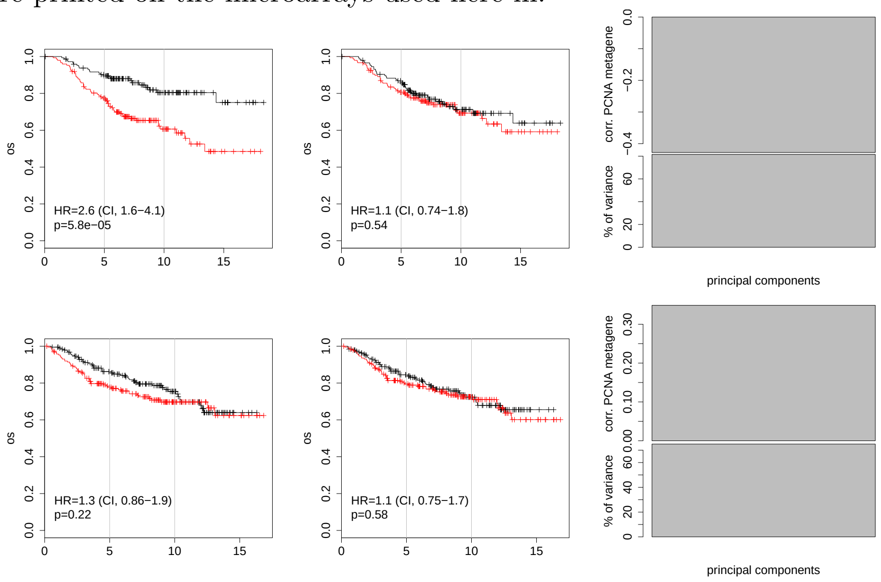

2.4 BEN-PORATH-EXP1

- Title of publication: An embryonic stem cell-like gene expression signature in poorly differentiated aggressive human tumors.
- Authors: I Ben-Porath MW Thomson VJ Carey R Ge GW Bell A Regev RA Weinberg
- Journal: Nat Genet
- Date of publication: May 2008
- PubMed ID: 18443585

367 genes symbols from this signature had aliases in Unigene. Among them, 330 are printed on the microarrays used here-in.

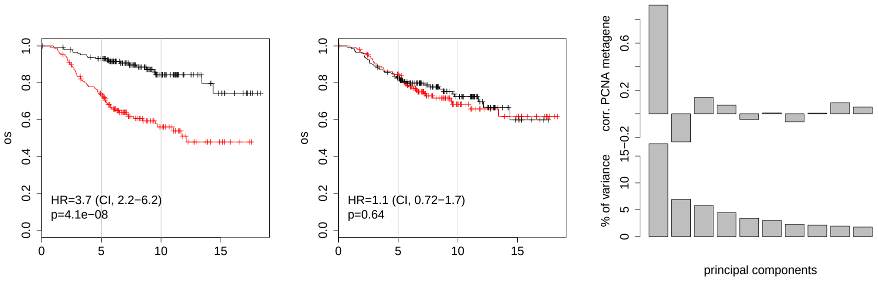

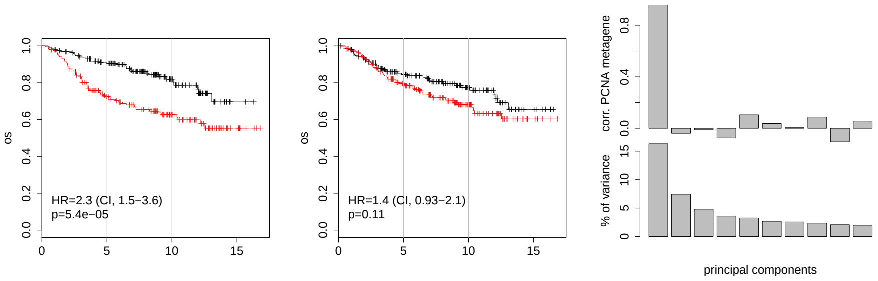

2.5 BEN-PORATH-PRC2

- Title of publication: An embryonic stem cell-like gene expression signature in poorly differentiated aggressive human tumors.
- Authors: I Ben-Porath MW Thomson VJ Carey R Ge GW Bell A Regev RA Weinberg
- Journal: Nat Genet
- Date of publication: May 2008
- PubMed ID: 18443585

631 genes symbols from this signature had aliases in Unigene. Among them, 463 are printed on the microarrays used here-in.

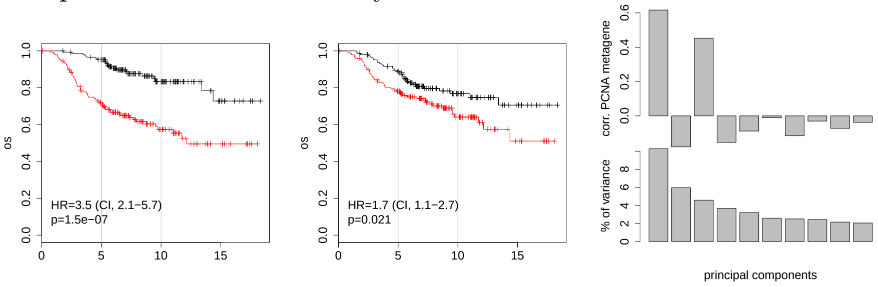

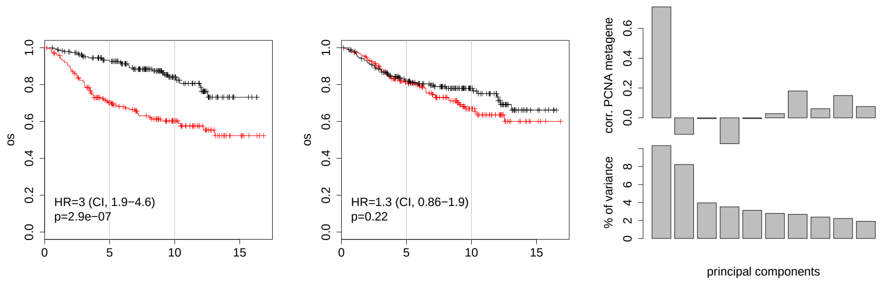

2.6 BUESS

- Title of publication: Characterization of heterotypic interaction effects in vitro to deconvolute global gene expression profiles in cancer.
- Authors: M Buess DS Nuyten T Hastie T Nielsen R Pesich PO Brown
- Journal: Genome Biol
- Date of publication: Month 2007
- PubMed ID: 17868458

30 genes symbols from this signature had aliases in Unigene. Among them, 24 are printed on the microarrays used here-in.

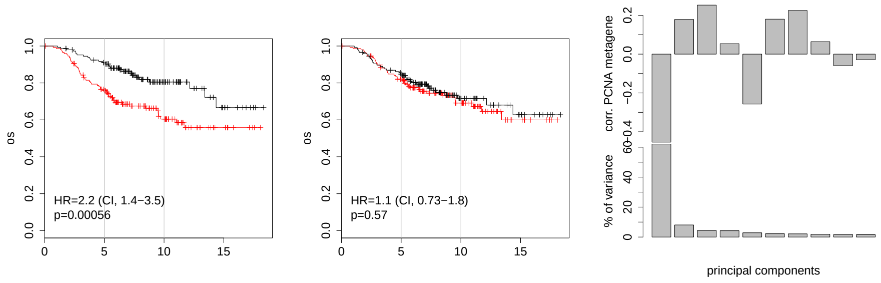

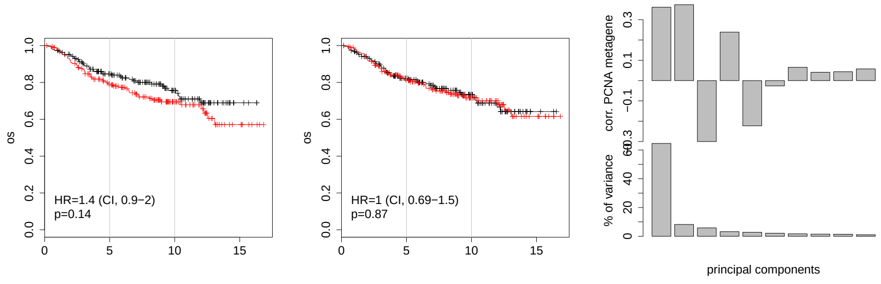

2.7 BUFFA

- Title of publication: Large meta-analysis of multiple cancers reveals a common, compact and highly prognostic hypoxia metagene.

- Authors: FM Buffa AL Harris CM West CJ Miller
- Journal: Br J Cancer
- Date of publication: Jan 2010
- PubMed ID: 20087356

3 genes symbols from this signature had aliases in Unigene. Among them, 2 are printed on the microarrays used here-in.

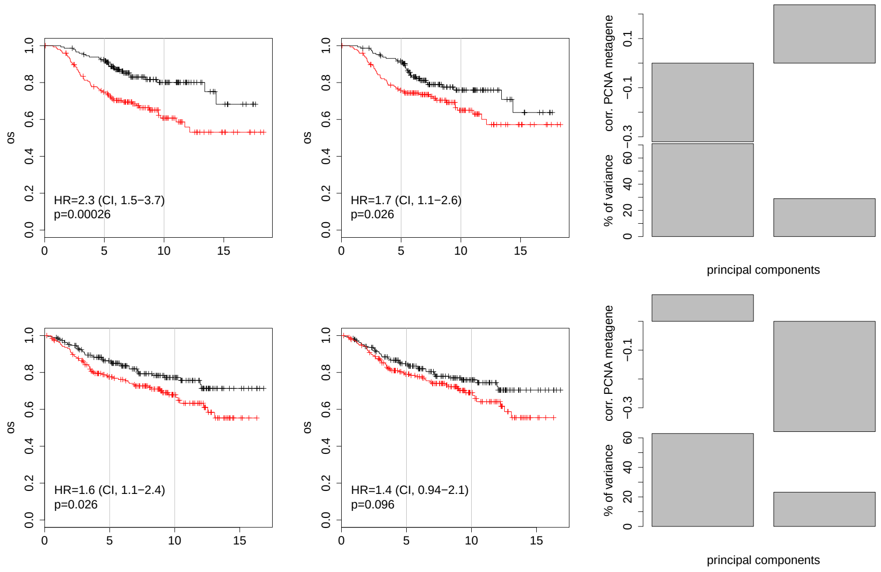

2.8 CARTER

- Title of publication: A signature of chromosomal instability inferred from gene expression profiles predicts clinical outcome in multiple human cancers.
- Authors: SL Carter AC Eklund IS Kohane LN Harris Z Szallasi
- Journal: Nat Genet
- Date of publication: Sep 2006
- PubMed ID: 16921376

70 genes symbols from this signature had aliases in Unigene. Among them, 69 are printed on the microarrays used here-in.

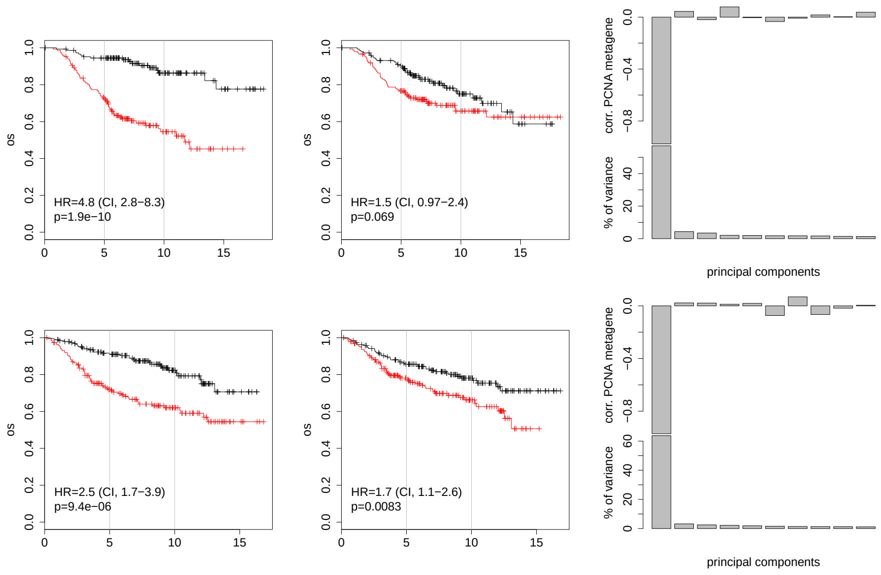

2.9 CHANG

- Title of publication: Gene expression signature of fibroblast serum response predicts human cancer progression: similarities between tumors and wounds.
- Authors: HY Chang JB Sneddon AA Alizadeh R Sood RB West K Montgomery JT Chi M van de Rijn D Botstein PO Brown
- Journal: PLoS Biol
- Date of publication: Feb 2004
- PubMed ID: 14737219

355 genes symbols from this signature had aliases in Unigene. Among them, 314 are printed on the microarrays used here-in.

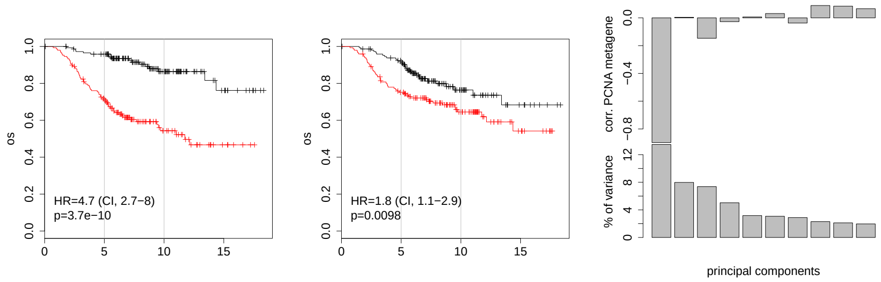

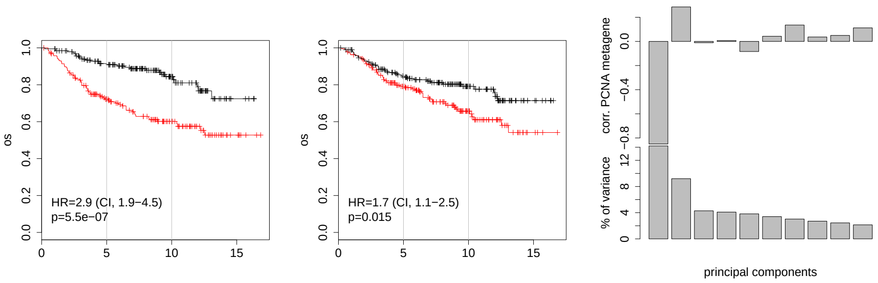

2.10 CHI

- Title of publication: Gene expression programs in response to hypoxia: cell type specificity and prognostic significance in human cancers.
- Authors: JT Chi Z Wang DS Nuyten EH Rodriguez ME Schaner A Salim Y Wang GB Kristensen A Helland AL Brresen-Dale A Giaccia MT Longaker T Hastie GP Yang MJ van de Vijver PO Brown
- Journal: PLoS Med
- Date of publication: Mar 2006
- PubMed ID: 16417408

136 genes symbols from this signature had aliases in Unigene. Among them, 113 are printed on the microarrays used here-in.

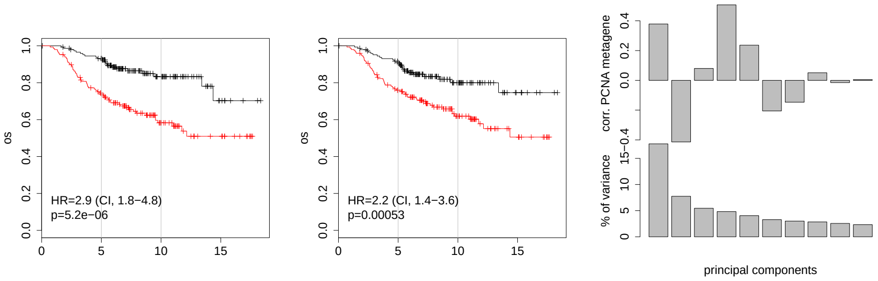

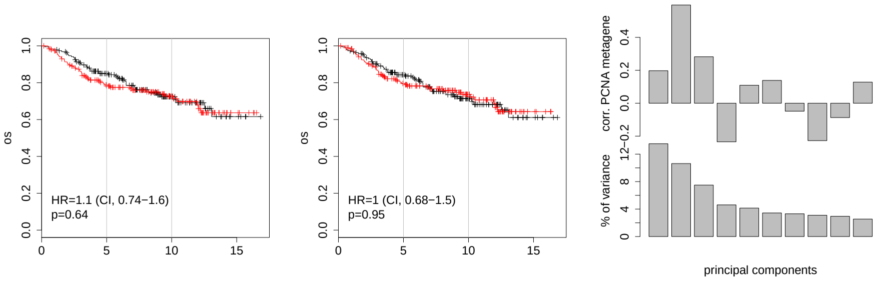

2.11 CRAWFORD

- Title of publication: Bromodomain 4 activation predicts breast cancer survival.
- Authors: NP Crawford J Alsarraj L Lukes RC Walker JS Officewala HH Yang MP Lee K Ozato KW Hunter
- Journal: Proc Natl Acad Sci U S A
- Date of publication: Apr 2008
- PubMed ID: 18427120

377 genes symbols from this signature had aliases in Unigene. Among them, 329 are printed on the microarrays used here-in.

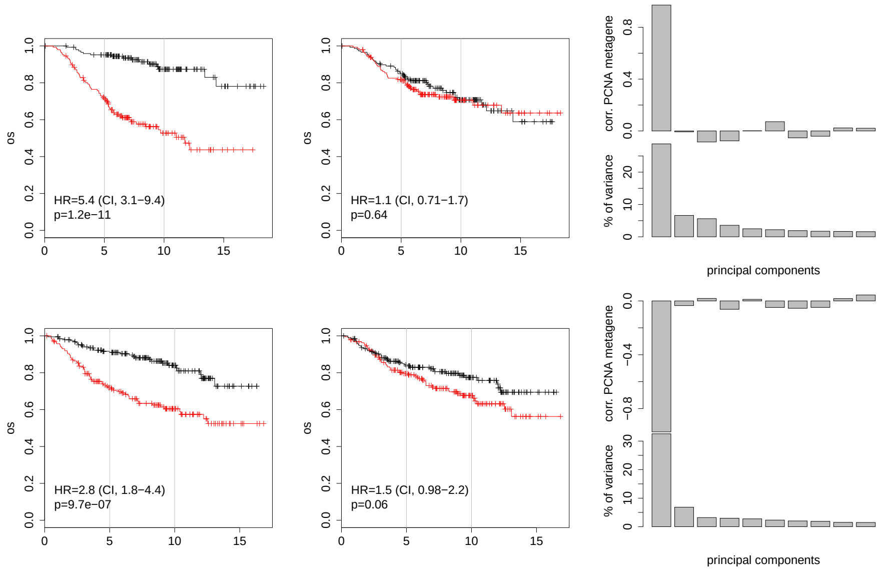

2.12 DAI

- Title of publication: A cell proliferation signature is a marker of extremely poor outcome in a subpopulation of breast cancer patients.
- Authors: H Dai L van't Veer J Lamb YD He M Mao BM Fine R Bernards M van de Vijver P Deutsch A Sachs R Stoughton S Friend
- Journal: Cancer Res
- Date of publication: May 2005
- PubMed ID: 15899795

35 genes symbols from this signature had aliases in Unigene. Among them, 35 are printed on the microarrays used here-in.

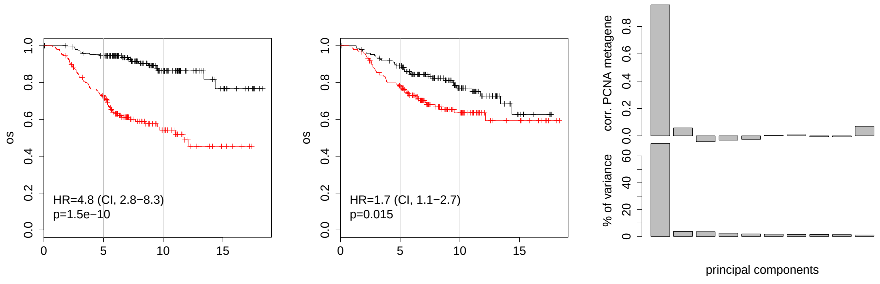

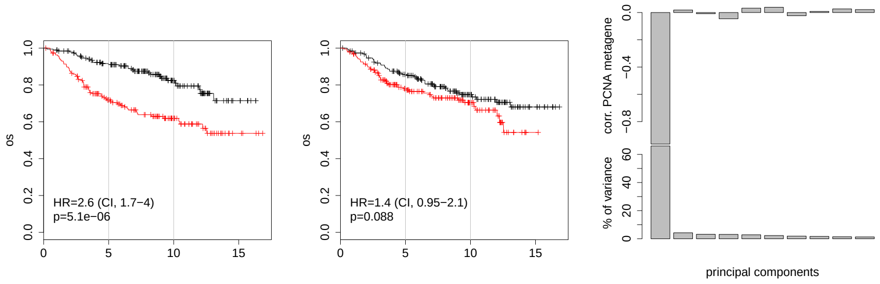

2.13 GLINSKY

- Title of publication: Microarray analysis identifies a death-from-cancer signature predicting therapy failure in patients with multiple types of cancer.
- Authors: GV Glinsky O Berezovska AB Glinskii
- Journal: J Clin Invest
- Date of publication: Jun 2005
- PubMed ID: 15931389

11 genes symbols from this signature had aliases in Unigene. Among them, 10 are printed on the microarrays used here-in.

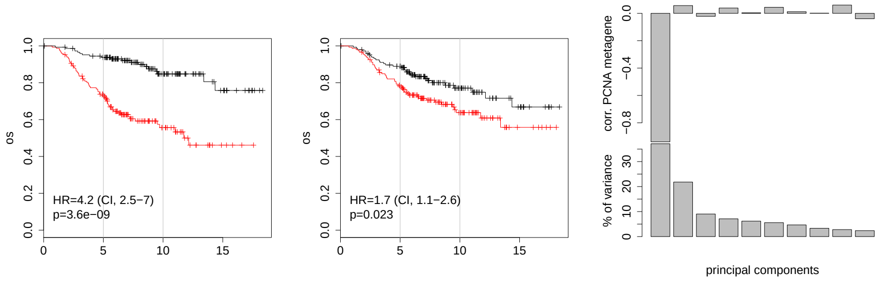

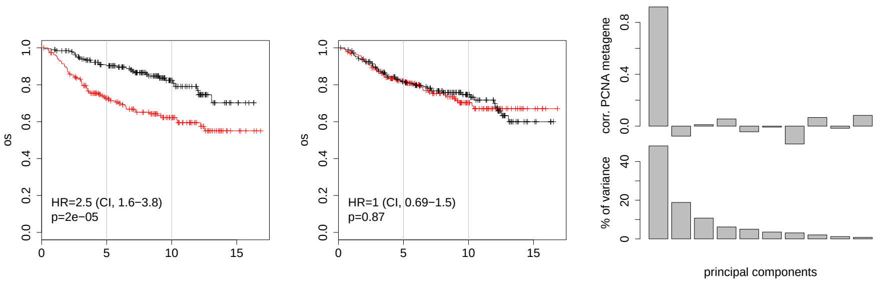

2.14 HALLSTROM

- Title of publication: An E2F1-dependent gene expression program that determines the balance between proliferation and cell death.
- Authors: TC Hallstrom S Mori JR Nevins
- Journal: Cancer Cell
- Date of publication: Jan 2008
- PubMed ID: 18167336

78 genes symbols from this signature had aliases in Unigene. Among them, 65 are printed on the microarrays used here-in.

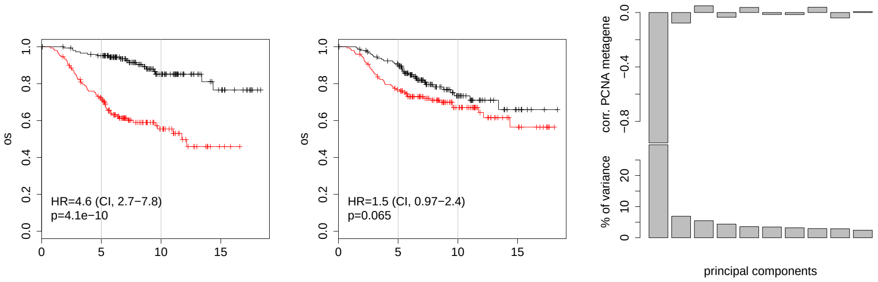

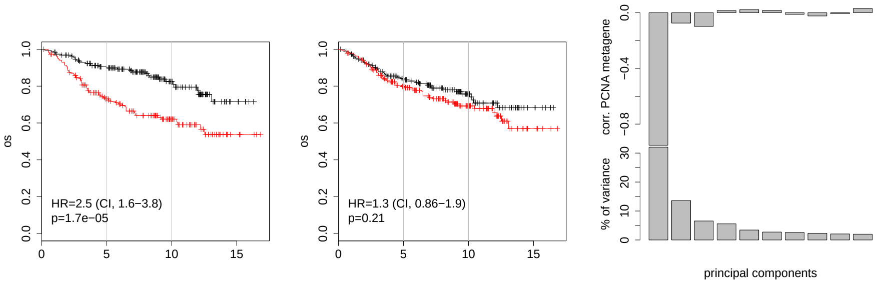

2.15 HE

- Title of publication: Expression signature developed from a complex series of mouse models accurately predicts human breast cancer survival.

- Authors: M He DP Mangiameli S Kachala K Hunter J Gillespie X Bian HC Shen SK Libutti
- Journal: Clin Cancer Res
- Date of publication: Jan 2010
- PubMed ID: 20028755

6 genes symbols from this signature had aliases in Unigene. Among them, 6 are printed on the microarrays used here-in.

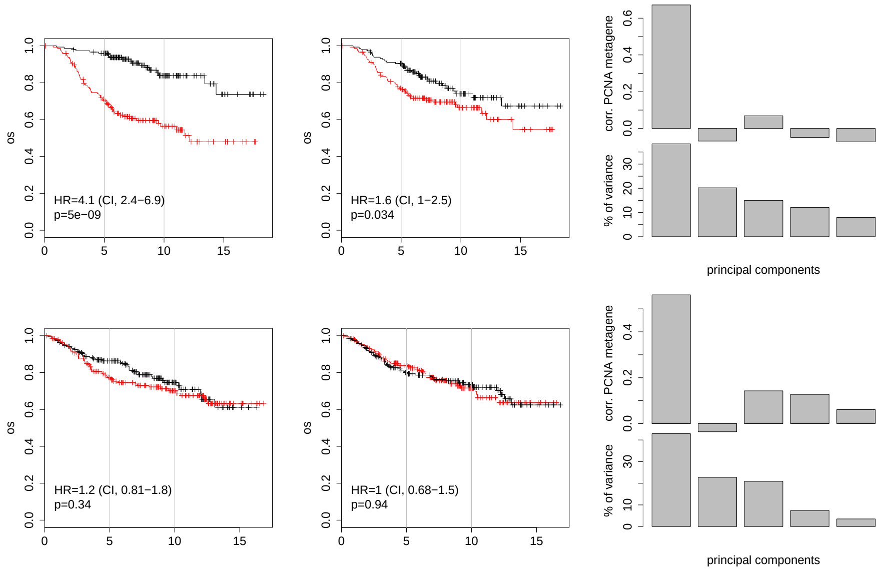

2.16 HU

- Title of publication: MTDH activation by 8q22 genomic gain promotes chemoresistance and metastasis of poor-prognosis breast cancer.
- Authors: G Hu RA Chong Q Yang Y Wei MA Blanco F Li M Reiss JL Au BG Haffty Y Kang
- Journal: Cancer Cell
- Date of publication: Jan 2009
- PubMed ID: 19111877

13 genes symbols from this signature had aliases in Unigene. Among them, 12 are printed on the microarrays used here-in.

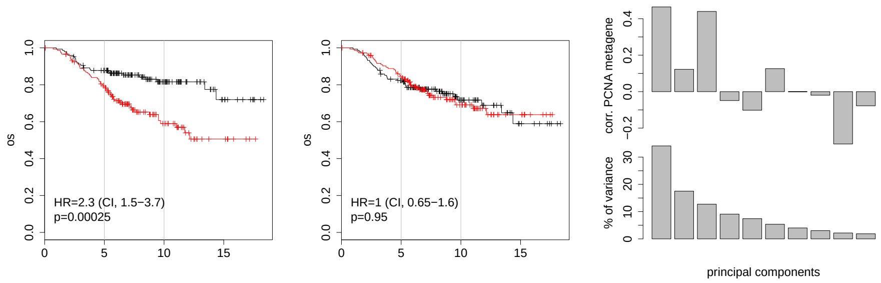

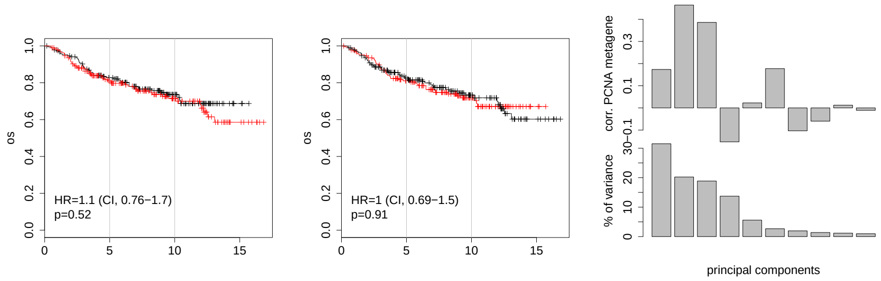

2.17 HUA

- Title of publication: Genomic antagonism between retinoic acid and estrogen signaling in breast cancer.
- Authors: S Hua R Kittler KP White
- Journal: Cell
- Date of publication: Jun 2009
- PubMed ID: 19563758

1345 genes symbols from this signature had aliases in Unigene. Among them, 1160 are printed on the microarrays used here-in.

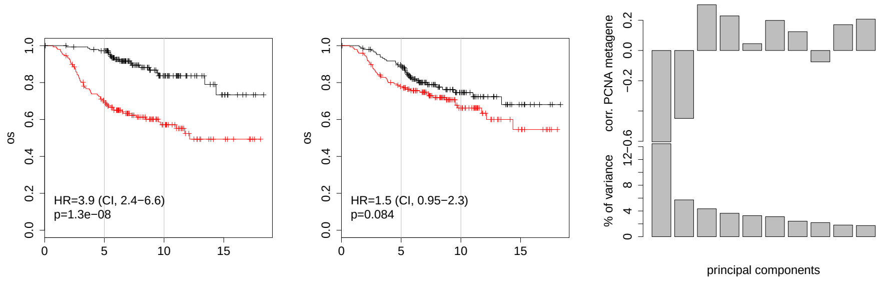

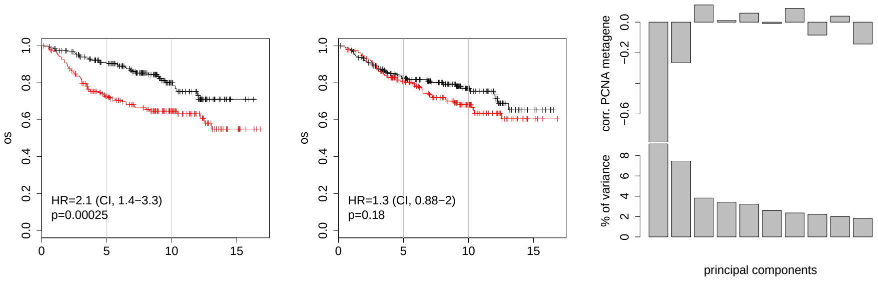

2.18 IVSHINA

- Title of publication: Genetic reclassification of histologic grade delineates new clinical subtypes of breast cancer.
- Authors: AV Ivshina J George O Senko B Mow TC Putti J Smeds T Lindahl Y Pawitan P Hall H Nordgren JE Wong ET Liu J Bergh VA Kuznetsov LD Miller
- Journal: Cancer Res
- Date of publication: Nov 2006
- PubMed ID: 17079448

17 genes symbols from this signature had aliases in Unigene. Among them, 14 are printed on the microarrays used here-in.

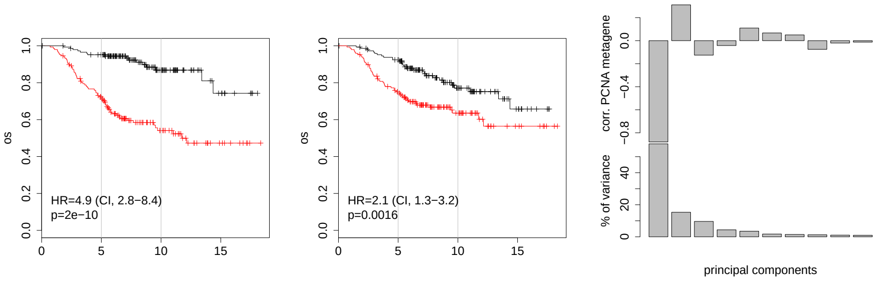

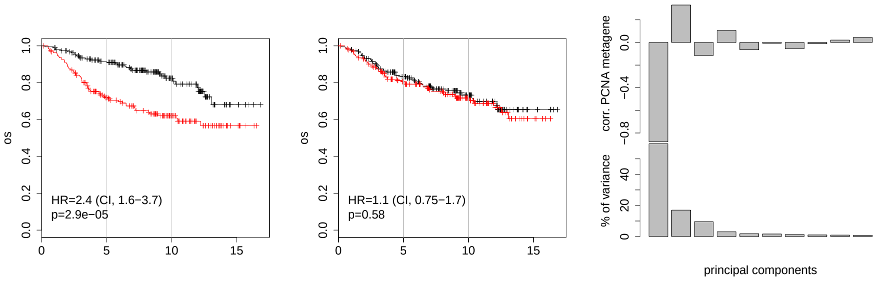

2.19 KOK

- Title of publication: Mammosphere-derived gene set predicts outcome in patients with ER-positive breast cancer.
- Authors: M Kok RH Koornstra TC Margarido R Fles NJ Armstrong SC Linn LJ Van't Veer B Weigelt
- Journal: J Pathol
- Date of publication: Jul 2009
- PubMed ID: 19353633

179 genes symbols from this signature had aliases in Unigene. Among them, 155 are printed on the microarrays used here-in.

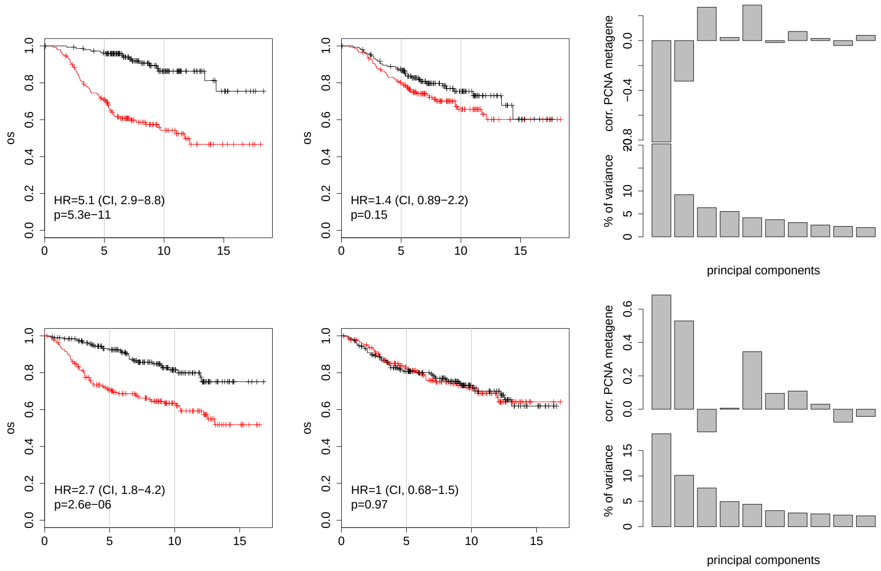

2.20 KORKOLA

- Title of publication: Identification of a robust gene signature that predicts breast cancer outcome in independent data sets.
- Authors: JE Korkola E Blaveri S DeVries DH Moore ES Hwang YY Chen AL Estep KL Chew RH Jensen FM Waldman
- Journal: BMC Cancer
- Date of publication: Month 2007
- PubMed ID: 17428335

21 genes symbols from this signature had aliases in Unigene. Among them, 20 are printed on the microarrays used here-in.

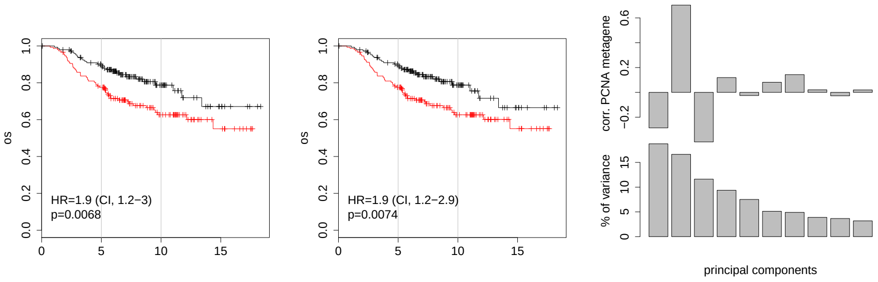

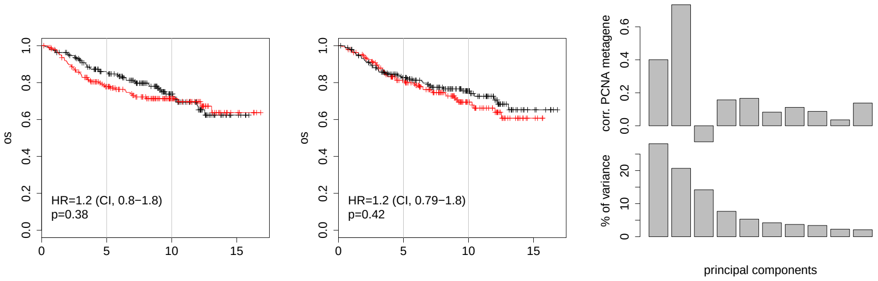

2.21 LIU

- Title of publication: The prognostic role of a gene signature from tumorigenic breast-cancer cells.
- Authors: R Liu X Wang GY Chen P Dalerba A Gurney T Hoey G Sherlock J Lewicki K Shedden MF Clarke
- Journal: N Engl J Med
- Date of publication: Jan 2007
- PubMed ID: 17229949

167 genes symbols from this signature had aliases in Unigene. Among them, 137 are printed on the microarrays used here-in.

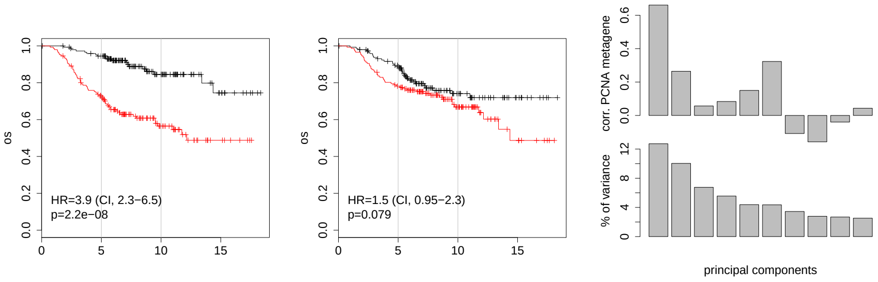

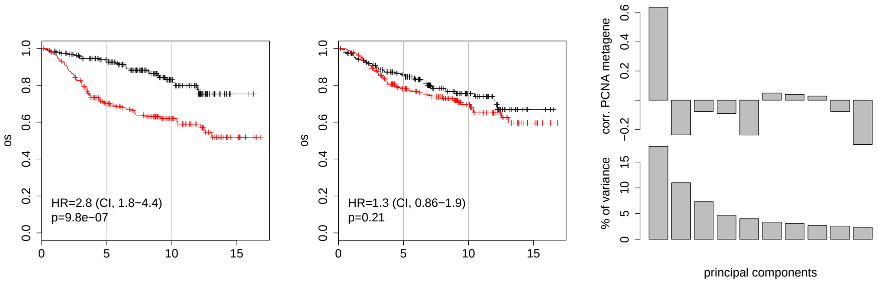

2.22 MA

- Title of publication: Gene expression profiles of human breast cancer progression.
- Authors: XJ Ma R Salunga JT Tuggle J Gaudet E Enright P McQuary T Payette M Pistone K Stecker BM Zhang YX Zhou H Varnholt B Smith M Gadd E Chatfield J Kessler TM Baer MG Erlander DC Sgroi
- Journal: Proc Natl Acad Sci U S A
- Date of publication: May 2003
- PubMed ID: 12714683

30 genes symbols from this signature had aliases in Unigene. Among them, 29 are printed on the microarrays used here-in.

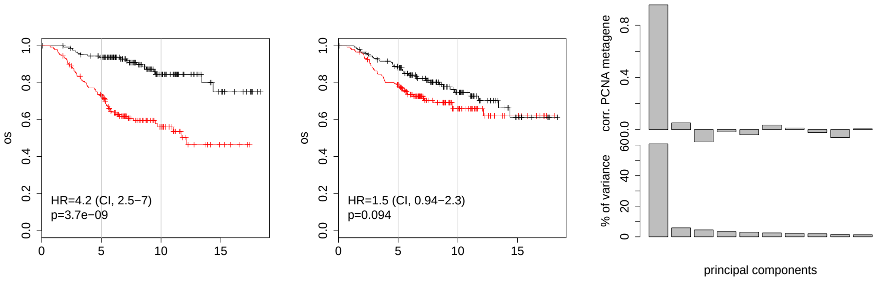

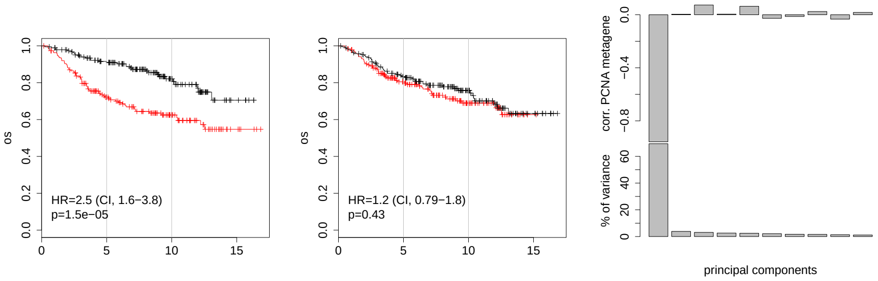

2.23 MILLER

- Title of publication: An expression signature for p53 status in human breast cancer predicts mutation status, transcriptional effects, and patient survival.
- Authors: LD Miller J Smeds J George VB Vega L Vergara A Ploner Y Pawitan P Hall S Klaar ET Liu J Bergh
- Journal: Proc Natl Acad Sci U S A
- Date of publication: Sep 2005
- PubMed ID: 16141321

18 genes symbols from this signature had aliases in Unigene. Among them, 17 are printed on the microarrays used here-in.

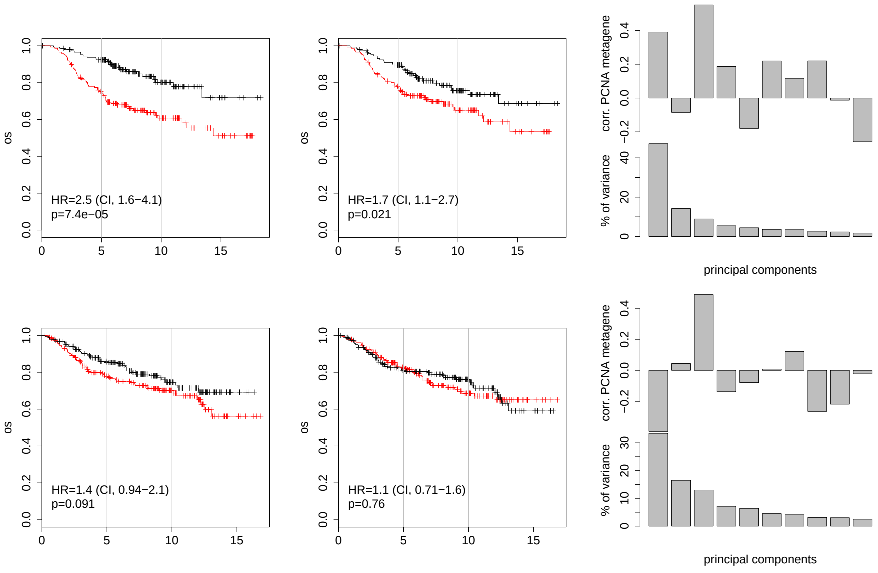

2.24 MORI

- Title of publication: Anchorage-independent cell growth signature identifies tumors with metastatic potential.
- Authors: S Mori JT Chang ER Andrechek N Matsumura T Baba G Yao JW Kim M Gatza S Murphy JR Nevins
- Journal: Oncogene
- Date of publication: Aug 2009
- PubMed ID: 19483725

156 genes symbols from this signature had aliases in Unigene. Among them, 122 are printed on the microarrays used here-in.

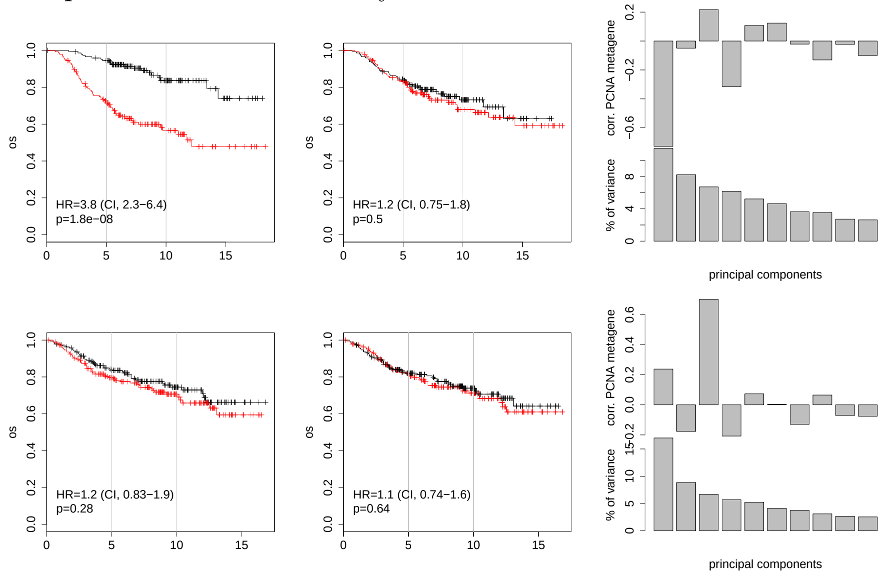

2.25 PAIK

- Title of publication: A multigene assay to predict recurrence of tamoxifen-treated, node-negative breast cancer.
- Authors: S Paik S Shak G Tang C Kim J Baker M Cronin FL Baehner MG Walker D Watson T Park W Hiller ER Fisher DL Wickerham J Bryant N Wolmark
- Journal: N Engl J Med
- Date of publication: Dec 2004
- PubMed ID: 15591335

16 genes symbols from this signature had aliases in Unigene. Among them, 16 are printed on the microarrays used here-in.

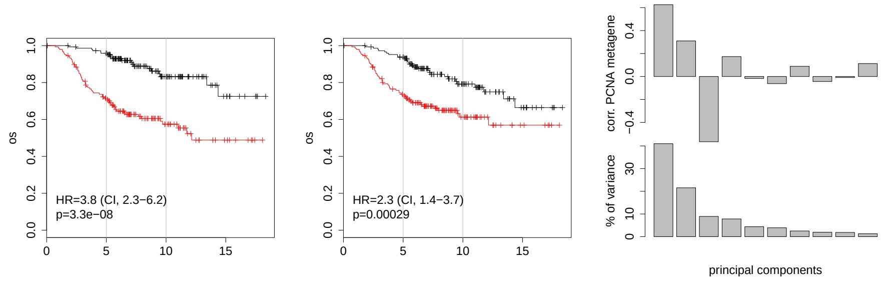

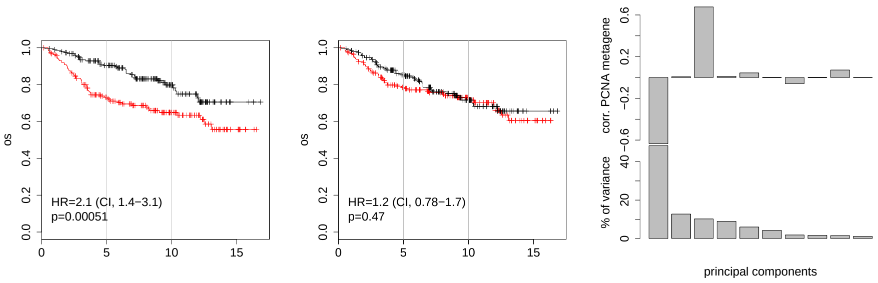

2.26 PAWITAN

- Title of publication: Intrinsic molecular signature of breast cancer in a population-based cohort of 412 patients.
- Authors: S Calza P Hall G Auer J Bjhle S Klaar U Kronenwett ET Liu L Miller A Ploner J Smeds J Bergh Y Pawitan
- Journal: Breast Cancer Res
- Date of publication: Month 2006
- PubMed ID: 16846532

46 genes symbols from this signature had aliases in Unigene. Among them, 41 are printed on the microarrays used here-in.

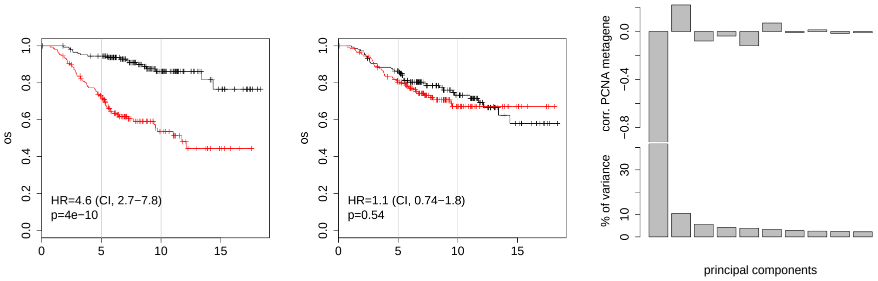

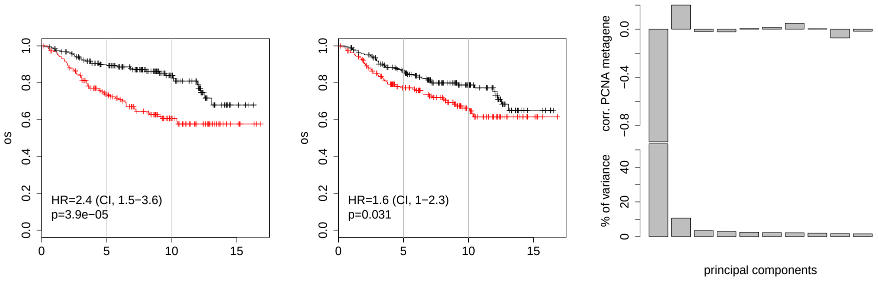

2.27 PEI

- Title of publication: CDK inhibitor p18(INK4c) is a downstream target of GATA3 and restrains mammary luminal progenitor cell prolif-

eration and tumorigenesis.

- Authors: XH Pei F Bai MD Smith J Usary C Fan SY Pai IC Ho CM Perou Y Xiong
- Journal: Cancer Cell
- Date of publication: May 2009
- PubMed ID: 19411068

2 genes symbols from this signature had aliases in Unigene. Among them, 2 are printed on the microarrays used here-in.

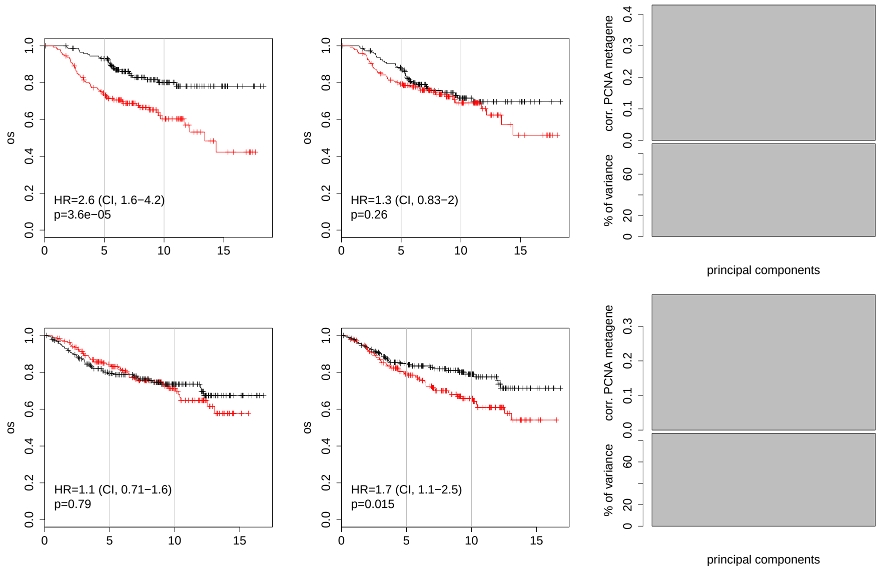

2.28 RAMASWAMY

- Title of publication: A molecular signature of metastasis in primary solid tumors.
- Authors: S Ramaswamy KN Ross ES Lander TR Golub
- Journal: Nat Genet
- Date of publication: Jan 2003
- PubMed ID: 12469122

16 genes symbols from this signature had aliases in Unigene. Among them, 14 are printed on the microarrays used here-in.

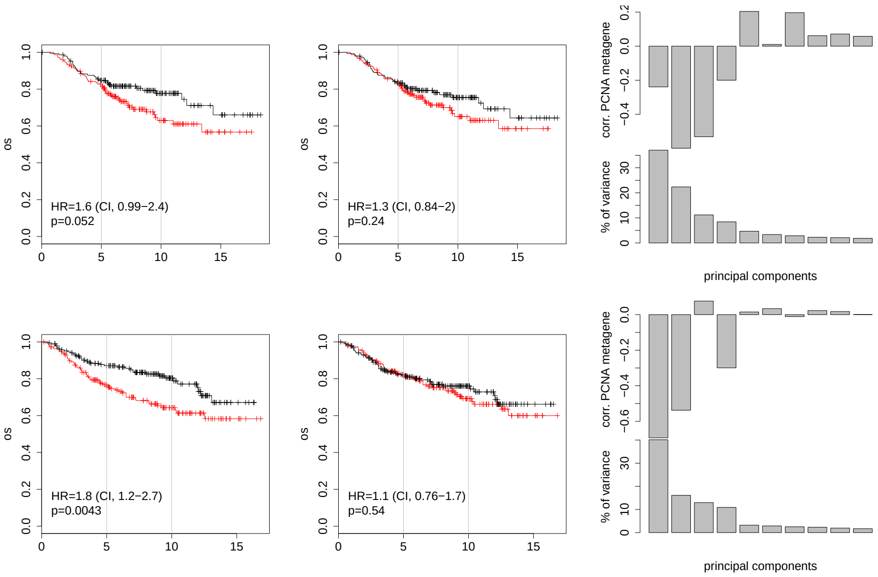

2.29 REUTER

- Title of publication: Modeling inducible human tissue neoplasia identifies an extracellular matrix interaction network involved in cancer progression.
- Authors: JA Reuter S Ortiz-Urda M Kretz J Garcia FA Scholl AM Pasmooij D Cassarino HY Chang PA Khavari
- Journal: Cancer Cell
- Date of publication: Jun 2009
- PubMed ID: 19477427

714 genes symbols from this signature had aliases in Unigene. Among them, 634 are printed on the microarrays used here-in.

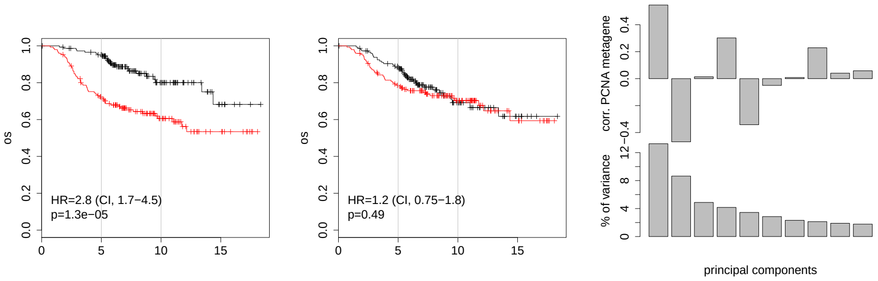

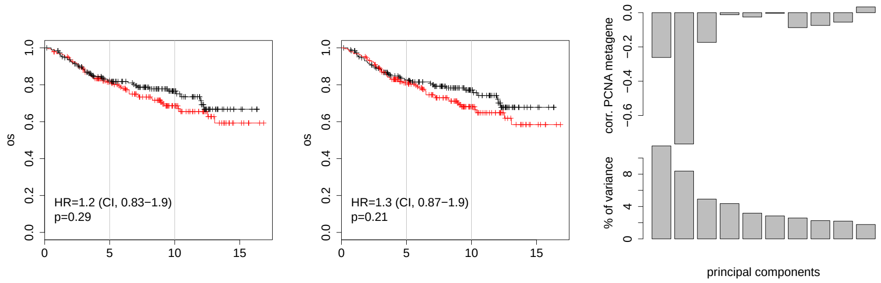

2.30 RHODES

- Title of publication: Large-scale meta-analysis of cancer microarray data identifies common transcriptional profiles of neoplastic transformation and progression.
- Authors: DR Rhodes J Yu K Shanker N Deshpande R Varambally D Ghosh T Barrette A Pandey AM Chinnaiyan
- Journal: Proc Natl Acad Sci U S A
- Date of publication: Jun 2004
- PubMed ID: 15184677

67 genes symbols from this signature had aliases in Unigene. Among them, 61 are printed on the microarrays used here-in.

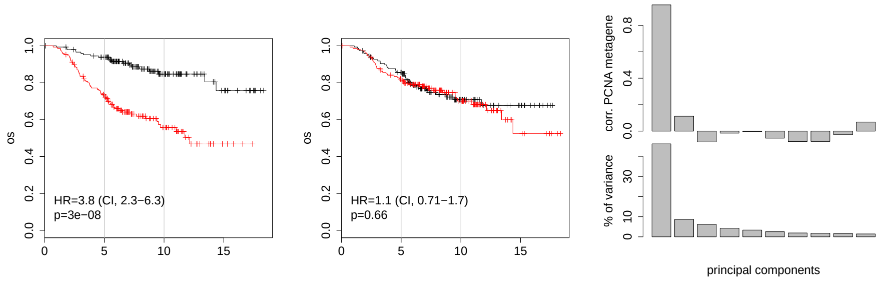

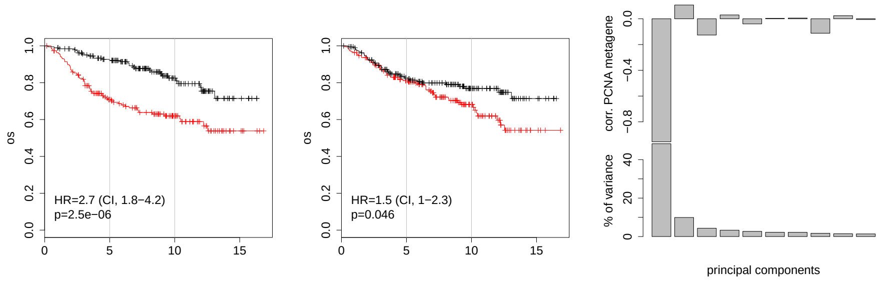

2.31 SAAL

- Title of publication: Poor prognosis in carcinoma is associated with a gene expression signature of aberrant PTEN tumor suppressor pathway activity.
- Authors: LH Saal P Johansson K Holm SK Gruvberger-Saal QB She M Maurer S Koujak AA Ferrando P Malmstrm L Memeo J Isola PO Bendahl N Rosen H Hibshoosh M Ringnr A Borg R Parsons
- Journal: Proc Natl Acad Sci U S A
- Date of publication: May 2007
- PubMed ID: 17452630

162 genes symbols from this signature had aliases in Unigene. Among them, 144 are printed on the microarrays used here-in.

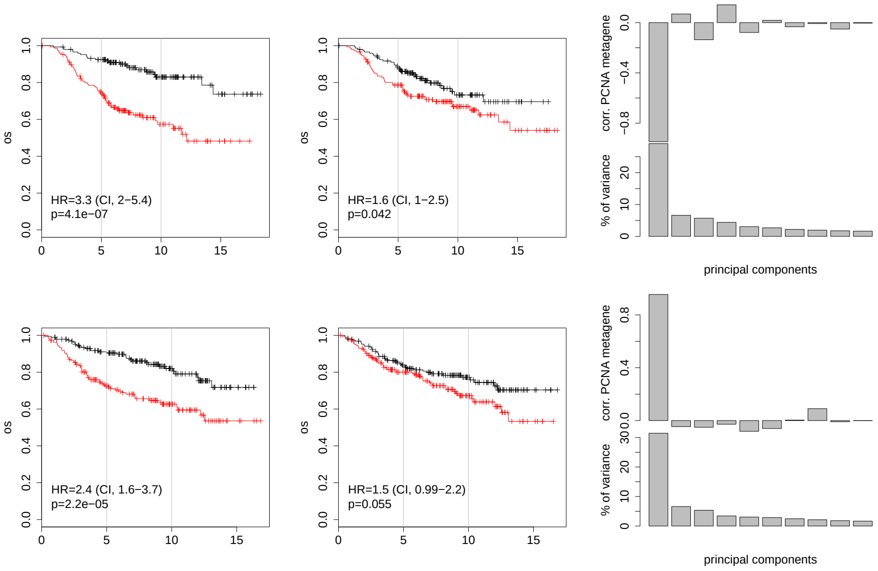

2.32 SHIPITSIN

- Title of publication: Molecular definition of breast tumor heterogeneity.
- Authors: M Shipitsin LL Campbell P Argani S Weremowicz N Bloushtain-Qimron J Yao T Nikolskaya T Serebryiskaya R Beroukhim M Hu MK Halushka S Sukumar LM Parker KS Anderson LN Harris JE Garber AL Richardson SJ Schnitt Y Nikolsky RS Gelman K Polyak
- Journal: Cancer Cell

- Date of publication: Mar 2007
- PubMed ID: 17349583

56 genes symbols from this signature had aliases in Unigene. Among them, 53 are printed on the microarrays used here-in.

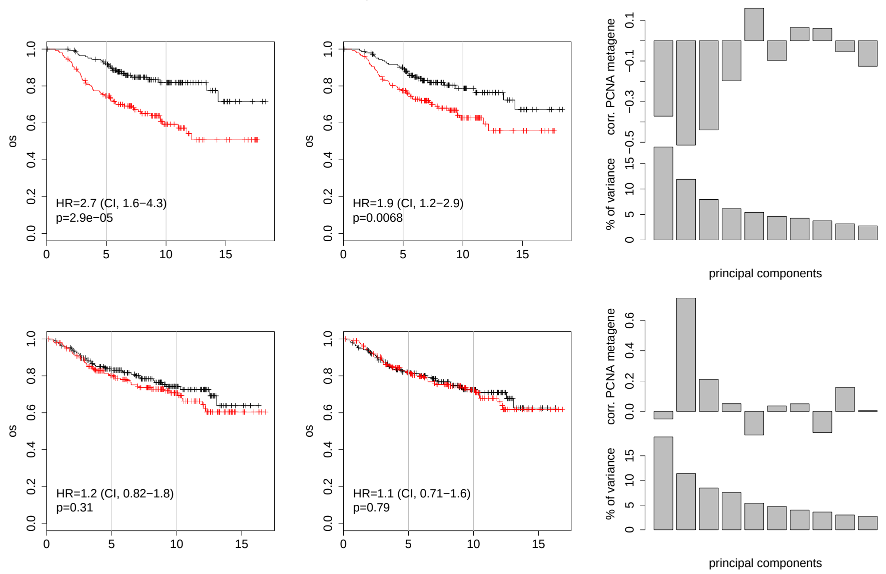

2.33 SORLIE

- Title of publication: Repeated observation of breast tumor subtypes in independent gene expression data sets.
- Authors: T Sorlie R Tibshirani J Parker T Hastie JS Marron A Nobel S Deng H Johnsen R Pesich S Geisler J Demeter CM Perou PE Lnning PO Brown AL Brresen-Dale D Botstein
- Journal: Proc Natl Acad Sci U S A
- Date of publication: Jul 2003
- PubMed ID: 12829800

15 genes symbols from this signature had aliases in Unigene. Among them, 14 are printed on the microarrays used here-in.

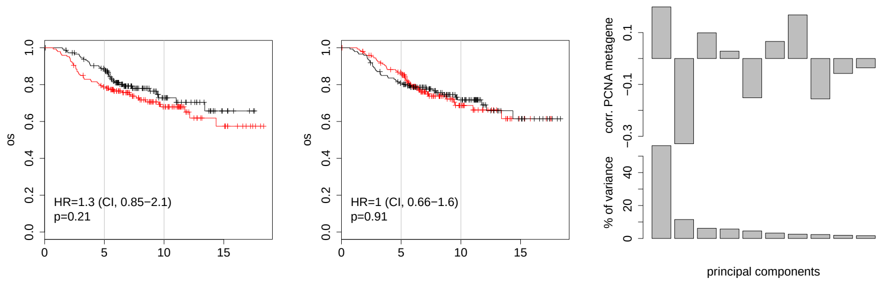

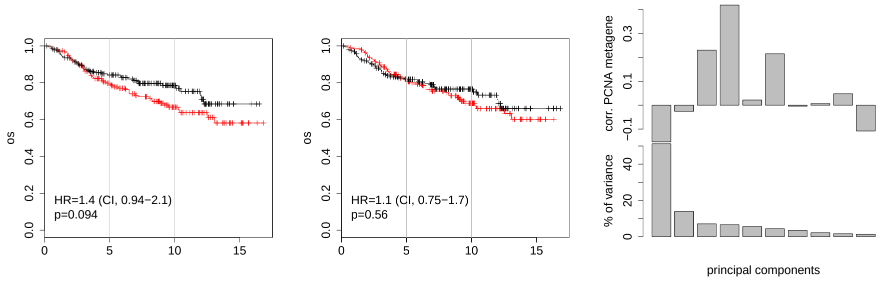

2.34 SOTIRIOU-93

- Title of publication: Breast cancer classification and prognosis based on gene expression profiles from a population-based study.
- Authors: C Sotiriou SY Neo LM McShane EL Korn PM Long A Jazaeri P Martiat SB Fox AL Harris ET Liu
- Journal: Proc Natl Acad Sci U S A
- Date of publication: Sep 2003
- PubMed ID: 12917485

343 genes symbols from this signature had aliases in Unigene. Among them, 317 are printed on the microarrays used here-in.

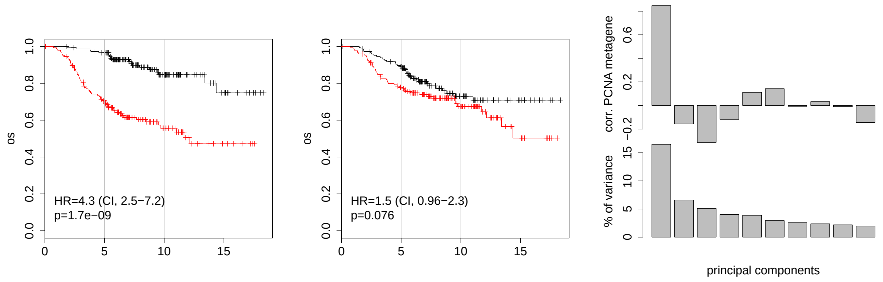

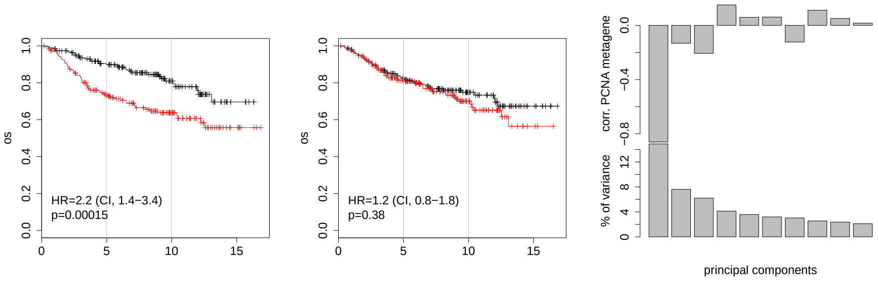

2.35 SOTIRIOU-GGI

- Title of publication: Gene expression profiling in breast cancer: understanding the molecular basis of histologic grade to improve prognosis.
- Authors: C Sotiriou P Wirapati S Loi A Harris S Fox J Smeds H Nordgren P Farmer V Praz B Haibe-Kains C Desmedt D Larsimont F Cardoso H Peterse D Nuyten M Buyse MJ Van de Vijver J Bergh M Piccart M Delorenzi
- Journal: J Natl Cancer Inst
- Date of publication: Feb 2006
- PubMed ID: 16478745

90 genes symbols from this signature had aliases in Unigene. Among them, 90 are printed on the microarrays used here-in.

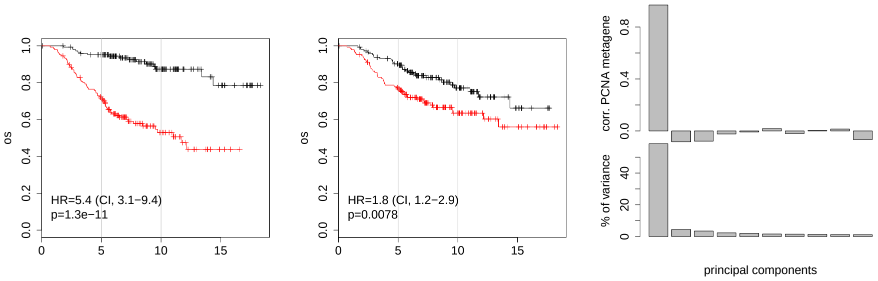

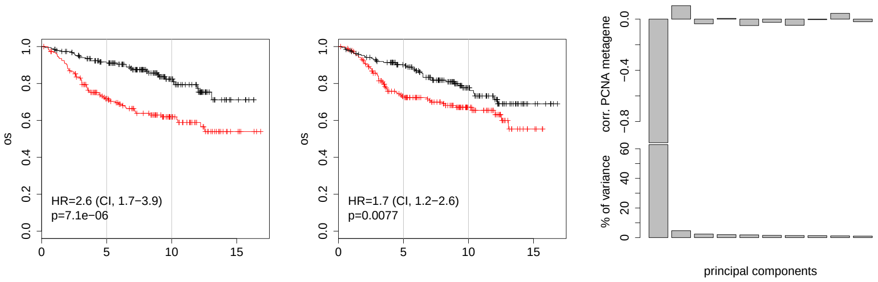

2.36 META-PCNA

This publication. 129 genes symbols from this signature had aliases in Unigene. Among them, 120 are printed on the microarrays used here-in.

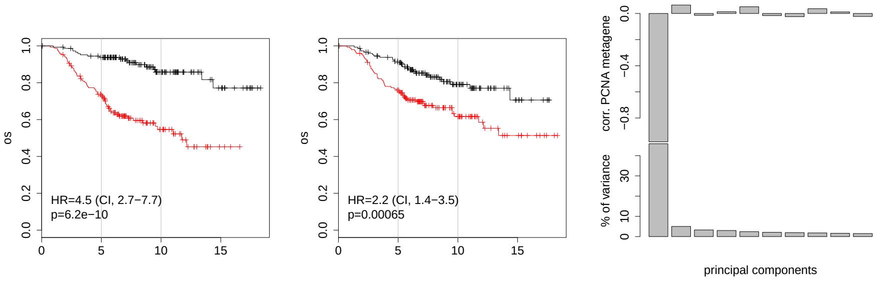

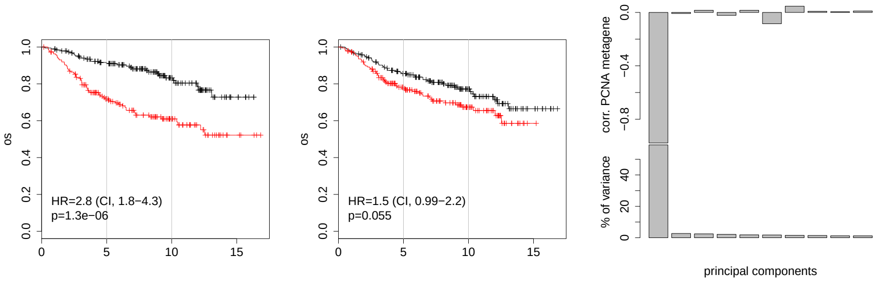

2.37 TAUBE

- Title of publication: Core epithelial-to-mesenchymal transition interactome gene-expression signature is associated with claudin-low and metaplastic breast cancer subtypes.
- Authors: JH Taube JI Herschkowitz K Komurov AY Zhou S Gupta J Yang K Hartwell TT Onder PB Gupta KW Evans BG Hollier PT Ram ES Lander JM Rosen RA Weinberg SA Mani
- Journal: Proc Natl Acad Sci U S A
- Date of publication: Aug 2010
- PubMed ID: 20713713

242 genes symbols from this signature had aliases in Unigene. Among them, 209 are printed on the microarrays used here-in.

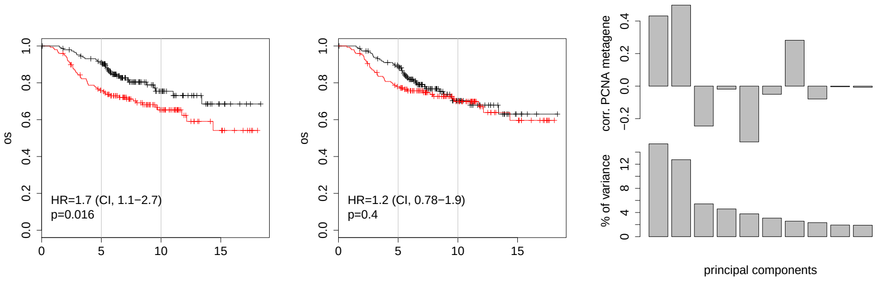

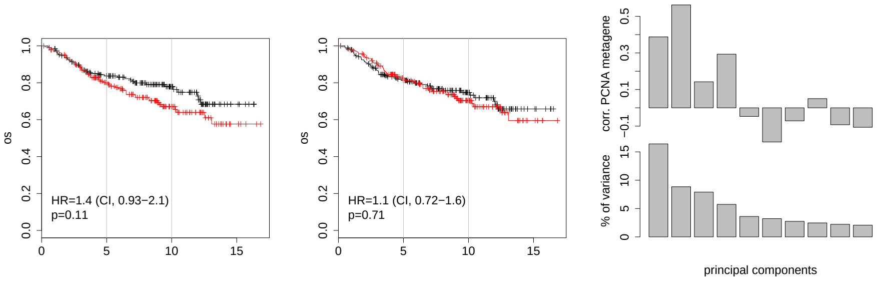

2.38 TAVAZOIE

- Title of publication: Endogenous human microRNAs that suppress breast cancer metastasis.
- Authors: SF Tavazoie C Alarcn T Oskarsson D Padua Q Wang PD Bos WL Gerald J Massagu
- Journal: Nature
- Date of publication: Jan 2008
- PubMed ID: 18185580

6 genes symbols from this signature had aliases in Unigene. Among them, 5 are printed on the microarrays used here-in.

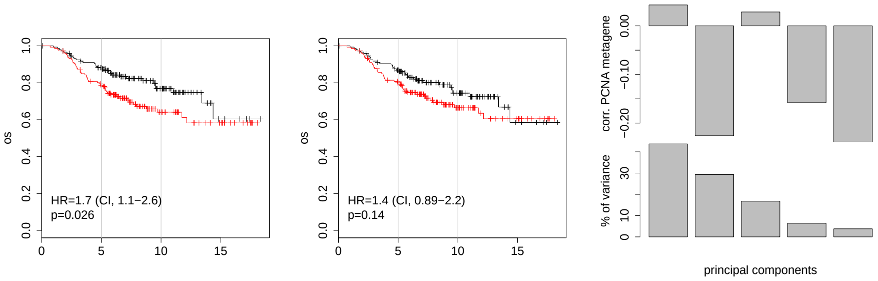

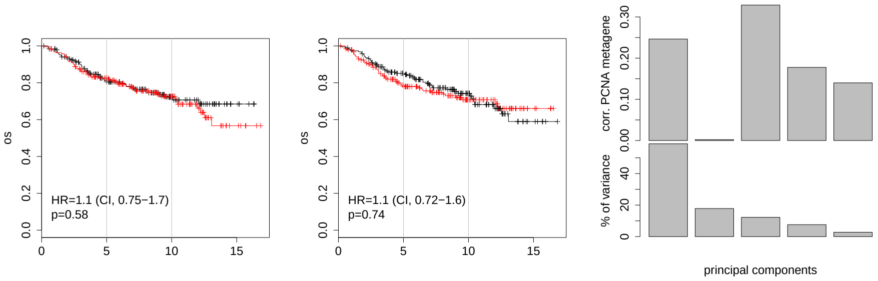

2.39 VALASTYAN

- Title of publication: A pleiotropically acting microRNA, miR-31, inhibits breast cancer metastasis.

- Authors: S Valastyan F Reinhardt N Benaich D Calogrias AM Szsz ZC Wang JE Brock AL Richardson RA Weinberg
- Journal: Cell
- Date of publication: Jun 2009
- PubMed ID: 19524507

6 genes symbols from this signature had aliases in Unigene. Among them, 6 are printed on the microarrays used here-in.

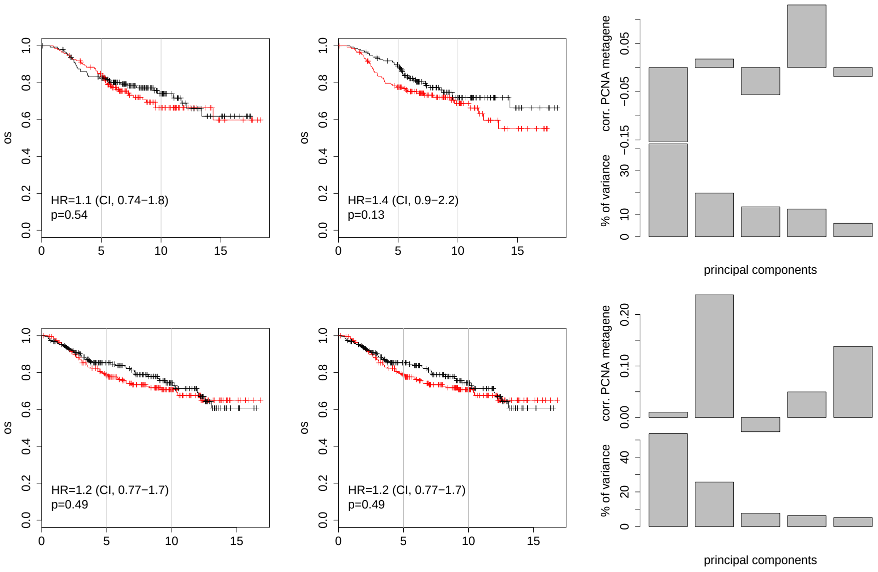

2.40 VANTVEER

- Title of publication: A gene-expression signature as a predictor of survival in breast cancer.
- Authors: MJ van de Vijver YD He LJ van't Veer H Dai AA Hart DW Voskuil GJ Schreiber JL Peterse C Roberts MJ Marton M Parrish D Atsma A Witteveen A Glas L Delahaye T van der Velde H Bartelink S Rodenhuis ET Rutgers SH Friend R Bernards
- Journal: N Engl J Med
- Date of publication: Dec 2002
- PubMed ID: 12490681

60 genes symbols from this signature had aliases in Unigene. Among them, 55 are printed on the microarrays used here-in.

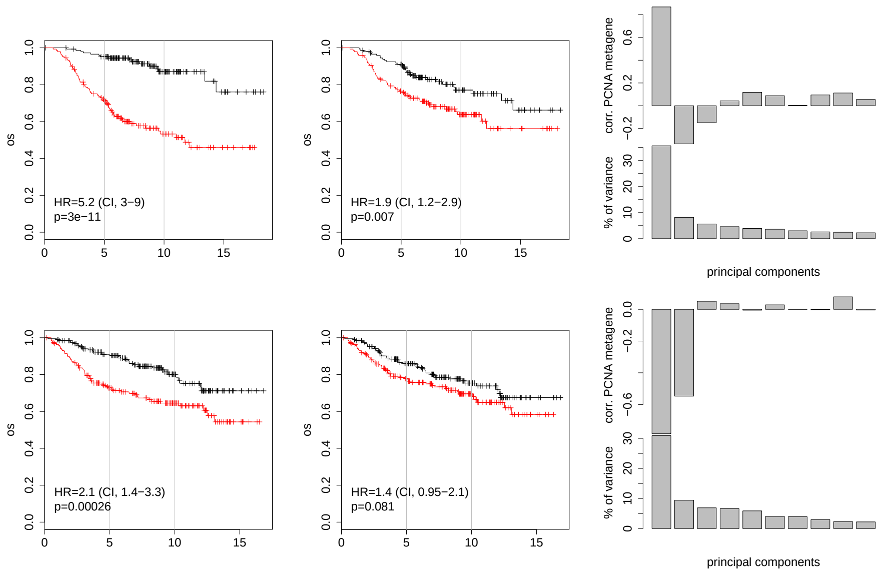

2.41 WANG-76

- Title of publication: Gene-expression profiles to predict distant metastasis of lymph-node-negative primary breast cancer.
- Authors: Y Wang JG Klijn Y Zhang AM Sieuwerts MP Look F Yang D Talantov M Timmermans ME Meijer-van Gelder J Yu T Jatkoe EM Berns D Atkins JA Foekens
- Journal: Lancet
- Date of publication: Month Year
- PubMed ID: 15721472

69 genes symbols from this signature had aliases in Unigene. Among them, 59 are printed on the microarrays used here-in.

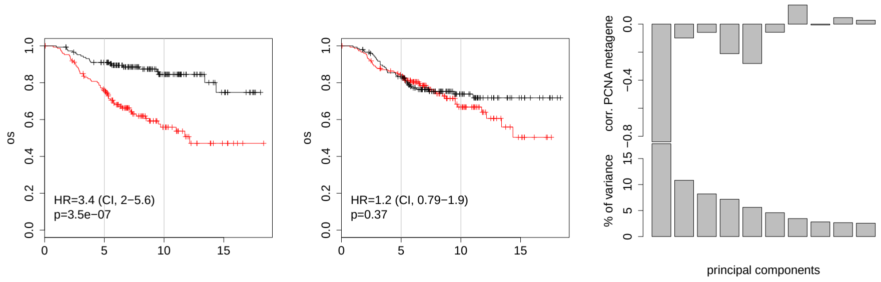

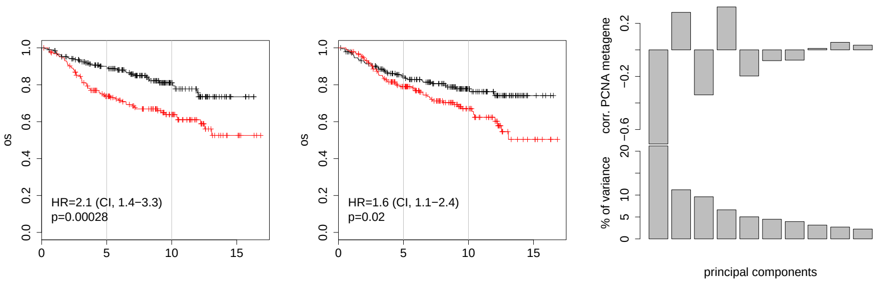

2.42 WANG-ALK5T204D

- Title of publication: Transforming growth factor beta engages TACE and ErbB3 to activate phosphatidylinositol-3 kinase/Akt in ErbB2-overexpressing breast cancer and desensitizes cells to trastuzumab.
- Authors: SE Wang B Xiang M Guix MG Olivares J Parker CH Chung A Pandiella CL Arteaga
- Journal: Mol Cell Biol
- Date of publication: Sep 2008
- PubMed ID: 18625725

239 genes symbols from this signature had aliases in Unigene. Among them, 188 are printed on the microarrays used here-in.

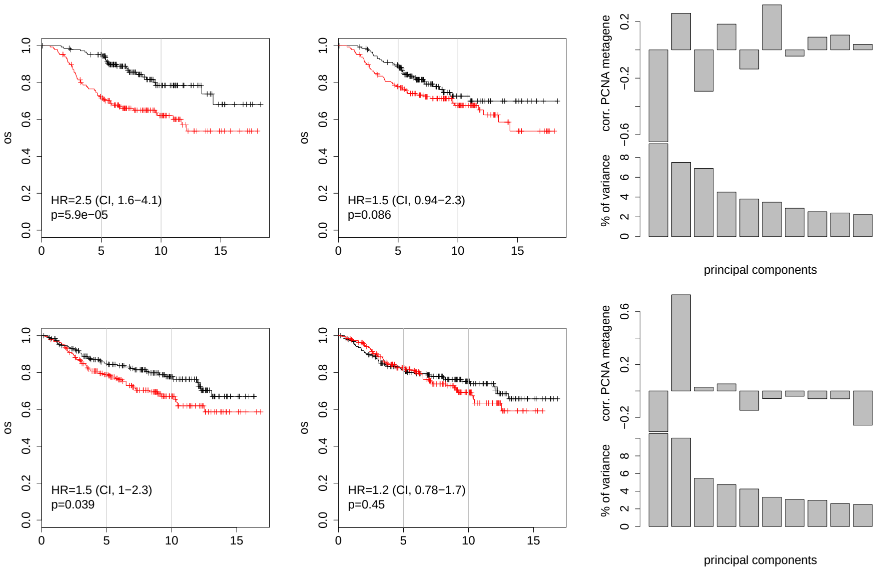

2.43 WELM

- Title of publication: The macrophage-stimulating protein pathway promotes metastasis in a mouse model for breast cancer and predicts poor prognosis in humans.
- Authors: AL Welm JB Sneddon C Taylor DS Nuyten MJ van de Vijver BH Hasegawa JM Bishop
- Journal: Proc Natl Acad Sci U S A
- Date of publication: May 2007
- PubMed ID: 17456594

3 genes symbols from this signature had aliases in Unigene. Among them, 3 are printed on the microarrays used here-in.

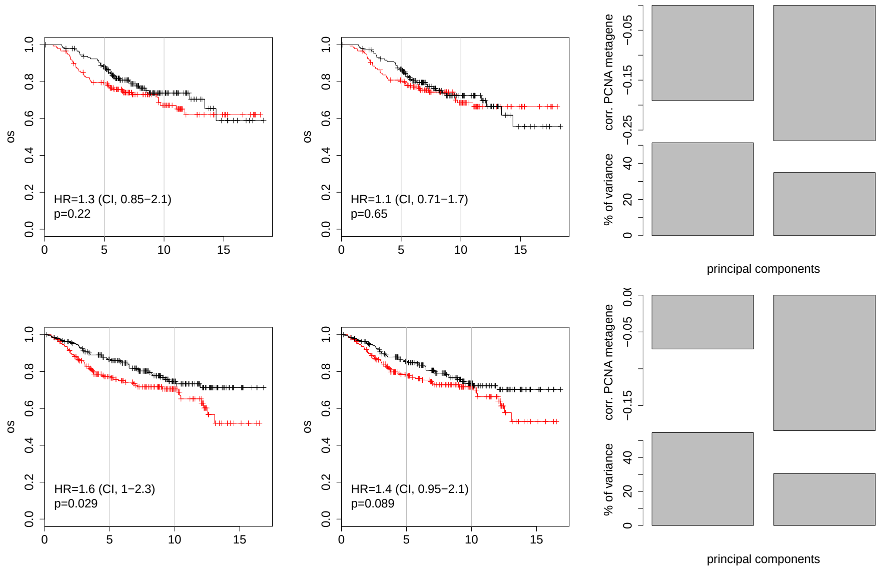

2.44 WEST

- Title of publication: Determination of stromal signatures in breast carcinoma.
- Authors: RB West DS Nuyten S Subramanian TO Nielsen CL Corless BP Rubin K Montgomery S Zhu R Patel T Hernandez-Boussard JR Goldblum PO Brown M van de Vijver M van de Rijn
- Journal: PLoS Biol
- Date of publication: Jun 2005
- PubMed ID: 15869330

468 genes symbols from this signature had aliases in Unigene. Among them, 402 are printed on the microarrays used here-in.

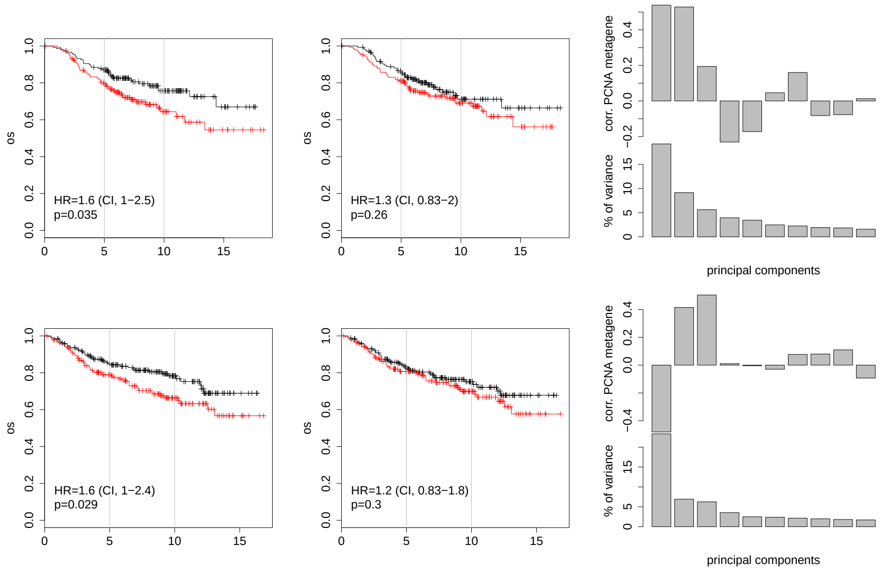

2.45 WHITFIELD

- Title of publication: Identification of genes periodically expressed in the human cell cycle and their expression in tumors.
- Authors: ML Whitfield G Sherlock AJ Saldanha JI Murray CA Ball KE Alexander JC Matese CM Perou MM Hurt PO Brown D Botstein
- Journal: Mol Biol Cell
- Date of publication: Jun 2002
- PubMed ID: 12058064

587 genes symbols from this signature had aliases in Unigene. Among them, 522 are printed on the microarrays used here-in.

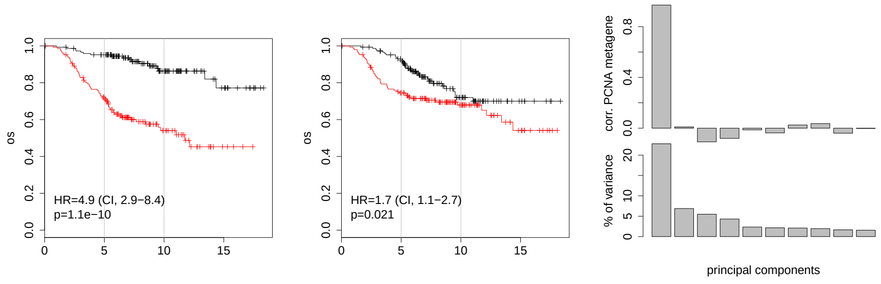

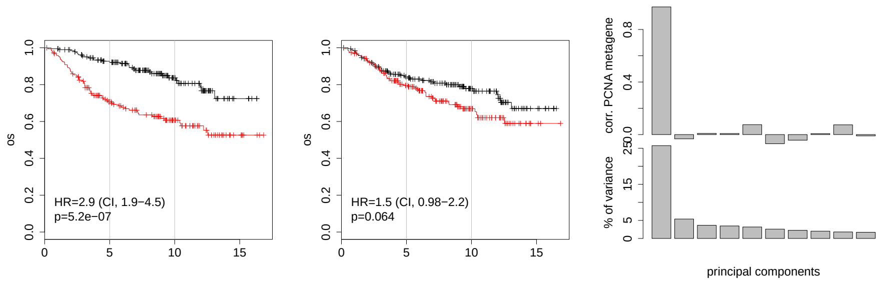

2.46 WONG-ESC

- Title of publication: Module map of stem cell genes guides creation of epithelial cancer stem cells.
- Authors: DJ Wong H Liu TW Ridky D Cassarino E Segal HY Chang
- Journal: Cell Stem Cell
- Date of publication: Apr 2008
- PubMed ID: 18397753

335 genes symbols from this signature had aliases in Unigene. Among them, 307 are printed on the microarrays used here-in.

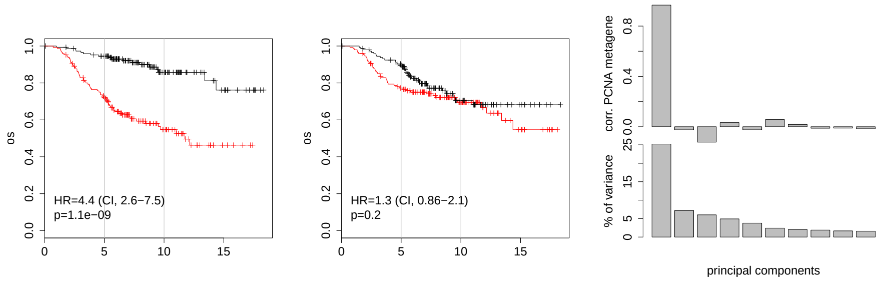

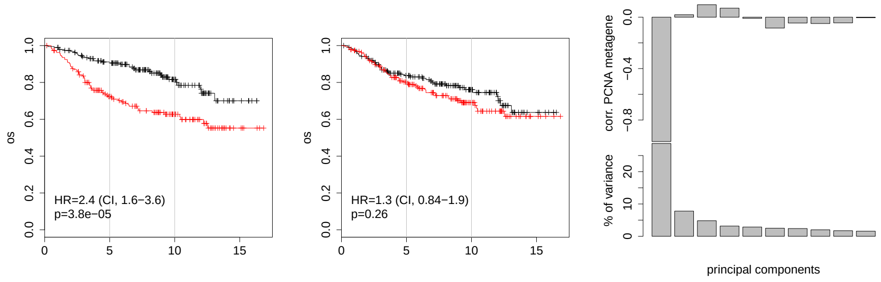

2.47 WONG-MITOCHON

- Title of publication: Revealing targeted therapy for human cancer by gene module maps.

- Authors: DJ Wong DS Nuyten A Regev M Lin AS Adler E Segal MJ van de Vijver HY Chang
- Journal: Cancer Res
- Date of publication: Jan 2008
- PubMed ID: 18199530

217 genes symbols from this signature had aliases in Unigene. Among them, 212 are printed on the microarrays used here-in.

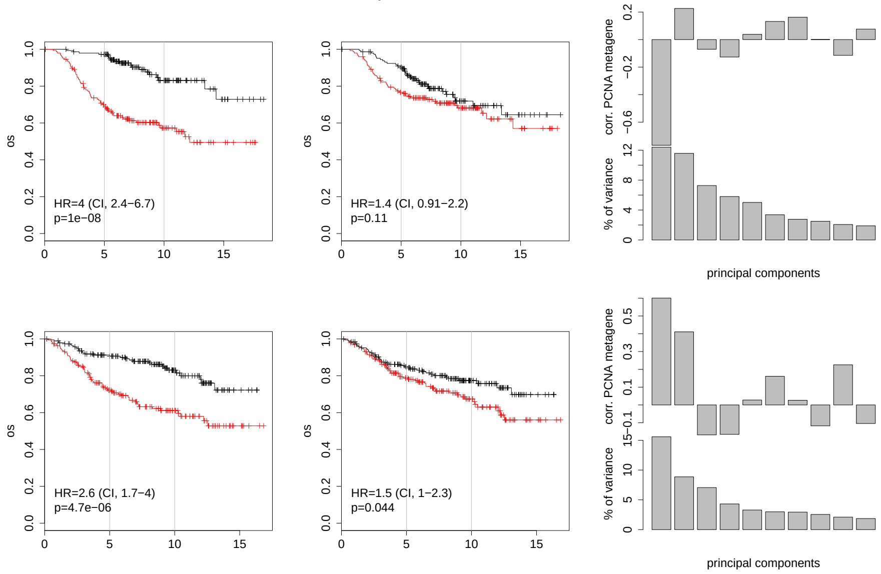

2.48 WONG-PROTEAS

- Title of publication: Revealing targeted therapy for human cancer by gene module maps.
- Authors: DJ Wong DS Nuyten A Regev M Lin AS Adler E Segal MJ van de Vijver HY Chang
- Journal: Cancer Res
- Date of publication: Jan 2008
- PubMed ID: 18199530

46 genes symbols from this signature had aliases in Unigene. Among them, 44 are printed on the microarrays used here-in.

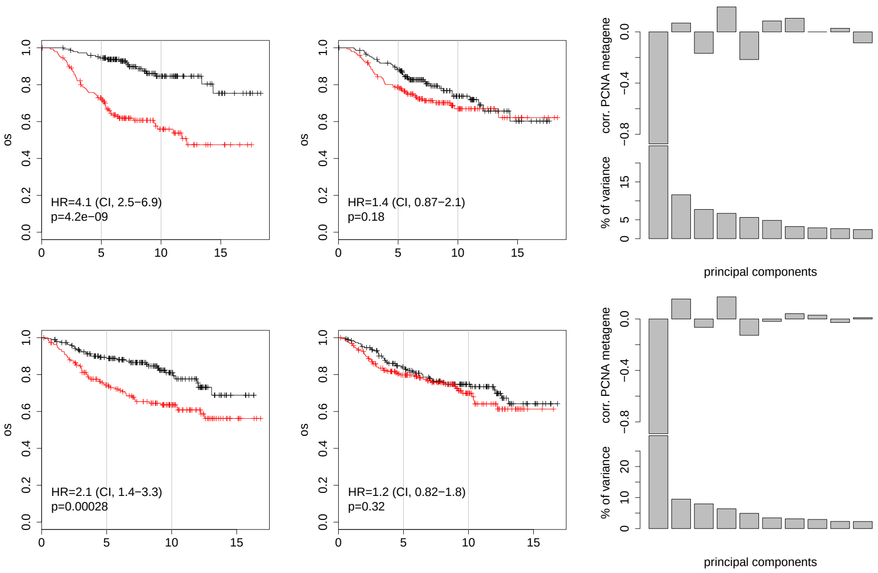

2.49 YU

- Title of publication: A polycomb repression signature in metastatic prostate cancer predicts cancer outcome.
- Authors: J Yu J Yu DR Rhodes SA Tomlins X Cao G Chen R Mehra X Wang D Ghosh RB Shah S Varambally KJ Pienta AM Chinnaiyan
- Journal: Cancer Res
- Date of publication: Nov 2007
- PubMed ID: 18006806

14 genes symbols from this signature had aliases in Unigene. Among them, 14 are printed on the microarrays used here-in.

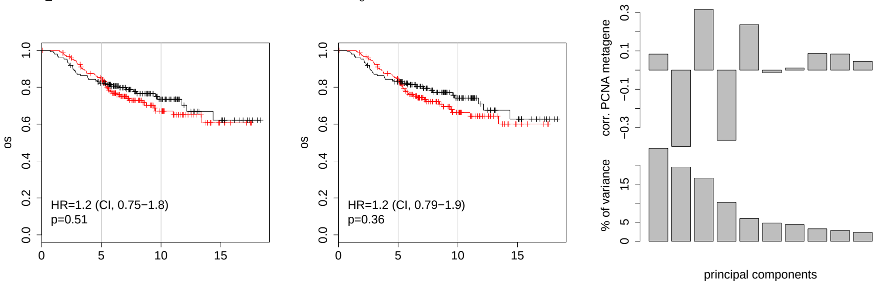

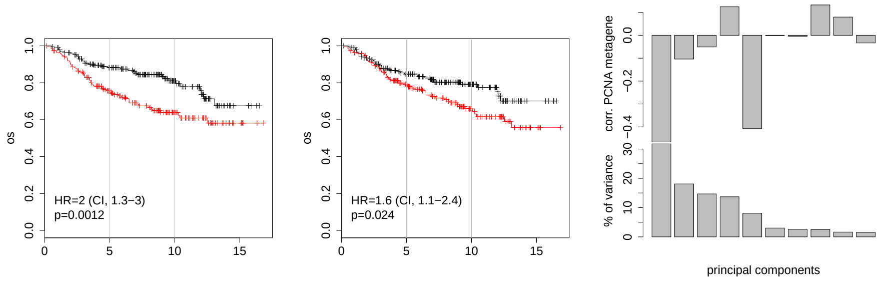

os
HR=2 (CI, 1.3−3)
p=0.0012
os
HR=1.6 (CI, 1.1−2.4)
p=0.024
corr. PCNA metagene
% of variance
principal components
